# Fire Intensity and spRead forecAst (FIRA): A Machine Learning Based Fire Spread Prediction Model for Air Quality Forecasting Application

**DOI:** 10.1029/2024GH001253

**Published:** 2025-03-22

**Authors:** Wei‐Ting Hung, Barry Baker, Patrick C. Campbell, Youhua Tang, Ravan Ahmadov, Johana Romero‐Alvarez, Haiqin Li, Jordan Schnell

**Affiliations:** ^1^ Air Resources Laboratory National Oceanic and Atmospheric Administration College Park MD USA; ^2^ Cooperative Institute for Satellite and Earth System Studies University of Maryland College Park MD USA; ^3^ Center for Spatial Information Science and Systems George Mason University Fairfax VA USA; ^4^ Global Systems Laboratory National Oceanic and Atmospheric Administration Boulder CO USA; ^5^ Cooperative Institute for Research in Environmental Sciences University of Colorado Boulder CO USA

**Keywords:** pollution: urban and regional, wildland fire model, monitoring, forecasting, prediction

## Abstract

Fire activities introduce hazardous impacts on the environment and public health by emitting various chemical species into the atmosphere. Most operational air quality forecast (AQF) models estimate smoke emissions based on the latest available satellite fire products, which may not represent real‐time fire behaviors without considering fire spread. Hence, a novel machine learning (ML) based fire spread forecast model, the Fire Intensity and spRead forecAst (FIRA), is developed for AQF model applications. FIRA aims to improve the performance of AQF models by providing realistic, dynamic fire characteristics including the spatial distribution and intensity of fire radiative power (FRP). In this study, data sets in 2020 over the continental United States (CONUS) and a historical California fire in 2024 are used for model training and evaluation. For application assessment, FIRA FRP predictions are applied to the Unified Forecast System coupled with smoke (UFS‐Smoke) model as inputs, focusing on a California fire case in September 2020. Results show that FIRA captures fire spread with R‐squared (*R* ^2^) near 0.7 and good spatial similarity (∼95%). The comparison between UFS‐Smoke simulations using near‐real‐time fire products and FIRA FRP predictions show good agreements, indicating that FIRA can accurately represent future fire activities. Although FIRA generally underestimates fire intensity, the uncertainties can be mitigated by applying scaling factors to FRP values. Use of the scaled FIRA largely outperforms the experimental UFS‐Smoke model in predicting aerosol optical depth and the three‐dimensional smoke contents, while also demonstrating the ability to improve surface fine particulate matter (PM_2.5_) concentrations affected by fires.

## Key Points

- A novel fire spread model that predicts fire spread and intensity is developed and evaluated for air quality forecasting applications
- Model predictions agree with satellite measurements and should provide near‐real‐time fire information to air quality forecasting models
- Model predictions are capable of improving air quality forecasting in predicting the three‐dimensional smoke contents and surface PM_2.5_

## 1 Introduction

Fire activities, including wildfires and agricultural burns, are one of the major sources of air pollutants in the United States (US) (Jaffe et al., 2020; Kaulfus et al., 2017; Larsen et al., 2018; Y. Li et al., 2021). The massive smoke emissions composed of fine particulate matter (PM_2.5_) and various trace gases (e.g., carbon monoxide (CO), methane (CH_4_), nitrogen oxides (NOx), and volatile organic compounds (VOCs)) have hazardous impacts on public health (Cascio, 2018; Jaffe et al., 2020; Kaulfus et al., 2017; Larsen et al., 2018; Ravi et al., 2018). Precursor emissions from fire activities undergo chemical reactions in the atmosphere and generate secondary air pollutants such as ozone (O_3_) and secondary organic aerosol (SOA), further introducing serious health concerns (McClure & Jaffe, 2018; Reid et al., 2019). Several health assessments stated that more than thousands of hospitalizations and premature deaths in the US, usually associated with respiratory diseases, could be attributed to smoke exposures (Fann et al., 2018; Holm et al., 2021; Ravi et al., 2018).

Air quality forecasting (AQF) is an efficient way to understand the potential health impacts of fire activities and to protect the public from harmful air pollutants. AQF modeling systems often incorporate the impact of fire occurrence and emissions on chemistry and air quality, such as the National Air Quality Forecasting Capability (NAQFC; Campbell et al., 2022; J. Huang et al., 2025; Y. Tang et al., 2022), the High‐Resolution Rapid Refresh coupled with Smoke (HRRR‐Smoke; Ahmadov et al., 2017), and the Aerosol Forecast Member in the Global Ensemble Forecast System (GEFS‐Aerosols; L. Zhang et al., 2022). These operational AQF models have the capability to simulate the fire behaviors, the burnt fuels, resulting emissions, rise and transport of smoke plumes, and the resulting air quality predictions and impacts.

In most AQF models that simulate the impacts of fire activities, the smoke emissions are estimated from fire size/intensity (i.e., the energy released from fires) and prescribed fuel‐specific emission factors (e.g., NAQFC and HRRR‐Smoke). Typically, the total smoke emission is first calculated based on fire intensity, usually from satellite‐based fire detection products such as fire radiative power (FRP), and then converted to the emission of a particular species using the ratio of emission factors of given species to a reference species like CO (Akagi et al., 2011; Andreae & Merlet, 2001; Kaiser et al., 2012).

In addition, fire behavior and spread (i.e., the spatiotemporal variations of fire activities) are critical to AQF in terms of predicting the future state of any given fire's duration, location, and emission allocation (Garcia‐Menendez et al., 2014). According to the Rothermel's surface fire spread model (Andrews, 2018; Rothermel, 1972), fire spread is correlated with fire intensity, fuel characteristics (e.g., the density, structure and moisture content of fuels), wind fields, and surface characteristics (e.g., terrain slope). It is a complex and highly variable process including various physical and dynamical phenomena across different spatiotemporal scales. Substantial time and computational resources are often required to parameterize and simulate the interactions between these phenomena in regional to global scale AQF models.

Certain advanced AQF models, such as the Weather Research and Forecasting (WRF) model coupled with Chemistry (WRF‐Chem; Grell et al., 2011; Powers et al., 2017) and the Wildfire Spread Simulation (SFIRE; Mandel et al., 2011) component, have the capability to explicitly simulate the fire behavior and spread and the implications for air quality at regional scales. However, such complicated and time consuming models usually have uncertainties in their underlying assumptions (e.g., fuel moisture and fuel loads), and are mostly used for retrospective fire cases at relatively limited scales (e.g., Kartsios et al., 2021; Kochanski et al., 2019; Y. Zhang et al., 2019), where such capabilities may not be feasible to be extended into the operational AQF community. While certain operational AQF models assume consistent fire growth with a static spread rate of burned area (e.g., the Canadian FireWork system (J. Chen et al., 2019)), most operational AQF modeling systems, such as the current NAQFC based on the Unified Forecast System‐Air Quality (UFS‐AQM) model (J. Huang et al., 2025) and HRRR‐Smoke at the National Oceanic and Atmospheric Administration (NOAA), do not account for fire behavior or spread due to the satellite‐based FRP framework, and limited time and computational resources for forecast applications. Additionally, the satellite FRP measurements are unavailable for the future forecasted times, and operational AQF models usually adopt the most recent available observations and assume mostly persistent, non‐spreading fires that introduce potential uncertainties into the air quality predictions.

To aid in fire‐related research and AQF applications, machine learning (ML) techniques have been widely used for fuel characterization and fire detection, as well as the predictions of fire weather, fire occurrence/risk, fire behaviors, and potential affects (Abid, 2021; Arif et al., 2021; Bot & Borges, 2022; Dampage et al., 2022; Jain et al., 2020). Several studies focused on fire spread (Allaire et al., 2021; Farguell et al., 2021; Hodges & Lattimer, 2019; X. Li, Zhang, Zhang, et al., 2022; Thapa et al., 2024; Zheng et al., 2017). For example, Hodges and Lattimer (2019) predicted the spatial evolution of wildland fires based on a deep convolutional neural network (CNN) model, using vegetative canopy characteristics such as vegetative canopy height and crown ratio, soil and fuel moistures at multiple layers, horizontal wind fields, and terrain elevation as training parameters. Results indicated that the CNN model could predict the spread of wildland fires up to 24 hr accurately, even for heterogeneous and complex terrains. Farguell et al. (2021) used a support vector machine (SVM) model to predict the propagation and the arrival time of fire fronts. Results were evaluated with the fire perimeters of 10 selected California fire incidents in 2020, introducing a good performance in the representation of fire spread. These studies well demonstrated ML techniques' capabilities in learning the spatiotemporal variabilities of fire activities and producing accurate prediction of future fire extent with lower computational cost. However, most studies trained ML models with fire perimeters and/or burned area for particular fire incidents, which are not readily available or applicable in near‐real‐time fire components in operational AQF modeling systems. Also, the evolution of future fire intensity is not considered in these ML models.

In this study, a novel ML‐based fire spread forecast model, the Fire Intensity and spRead forecAst (FIRA), is developed for AQF model applications. The goal is to improve the performance of AQF models by replacing the assumed persistent fires from prior days with more realistic, dynamic information of future fire activities including both fire spread and intensity. FIRA focuses on the propagation of existing fires and does not include new ignitions. Two ML models for the predictions of fire intensity and spread, and a forecast workflow are developed and evaluated using a full‐year data from 2020. The summer of 2020 is marked as a historic wildfire season in California with severe fire incidents from mid‐August to early October, including the August Complex Fire and the Creek Fire (Keeley & Syphard, 2021; Varga et al., 2022). A wildfire case on 20 August 2020, an agricultural fire case on 4 August 2020, are selected for model evaluation. The historical California Park Fire on 26 July 2024, is also analyzed to evaluate the ML models' temporal generalizability, namely the capability to generate reliable predictions for periods other than 2020. Furthermore, the Unified Forecast System coupled with smoke (UFS‐Smoke; Ahmadov et al., 2022) model developed at NOAA is used as an example of a smoke forecast model. The impacts of FIRA modulated smoke emissions on air quality during September 7th–10th, 2020 are simulated by UFS‐Smoke for the assessment of AQF modeling application.

This manuscript is structured as follows. The data sets used in this study, the configurations and structures of the ML models and the forecast workflow, and the statistical analysis approaches are described in Section 2. Evaluations of the ML models and the forecast workflow, and results of variable importance analysis are discussed in Section 3. An assessment of AQF applications based on UFS‐Smoke simulations, including the comparison of UFS‐Smoke results using near‐real‐time satellite‐based FRP products and FIRA FRP predictions in terms of total‐column and three‐dimensional smoke contents, is given in Section 4. Major sources of model uncertainties and limitations are discussed in Section 5, with overall study conclusions in Section 6.

## 2 Methodology

### 2.1 Data Sets

Table 1 summarizes all the data sets used in this study. The ML models are trained and evaluated based on a 1‐year data set of 2020 over the continental United States (CONUS). Twelve variables are used as inputs for ML model training and forecasting, including FRP, meteorological parameters such as 2‐m temperature (T), 2‐m specific humidity (SH), 10‐m wind speed and direction (WS and WD) and precipitation rate (PRATE), fuel parameters such as surface type (ST), vegetation health index (VHI) and forest height (FH), terrain elevation (ELV), day of year (DOY), and hour of the day in local time (HOUR). Distribution plots of the 12 input variables are shown in Appendix 1 in Supporting Information S1. Geographic information (latitude and longitude) is used for location identification of fire predictions generated from the ML models in the mapping process, and is not included in the training and forecasting processes.

**Table 1:** List of Data Sets Used in This Study

Application	Data set	Source	Spatial/Temporal resolution	Data period
ML model training and forecasting	Fire radiative power	RAVE^a^	3 km/Hourly	2020
	2‐m temperature	HRRRv3^b^	3 km/Hourly	2020
	2‐m specific humidity	HRRRv3	3 km/Hourly	2020
	Precipitation rate	HRRRv3	3 km/Hourly	2020
	10‐m wind speed	HRRRv3	3 km/Hourly	2020
	10‐m wind direction	HRRRv3	3 km/Hourly	2020
	Surface type	VIIRS AST^c^	1 km/Annual	2020
	Vegetation health index	VIIRS VHP^d^	1 km/Weekly	2020
	Forest height	GEDI + Landsat^e^	30 m/Annual	2019–2020 (GEDI) 1997–2020 (Landsat)
	Terrain elevation	Yamazaki et al. (2017)^f^	0.25°/Climatology	2000, 2006–2011
	Day of year	–	–	2020
	Hour of the day	–	–	2020
Geolocation mapping	Latitude	RAVE	3 km/‐	2020
	Longitude	RAVE	3 km/‐	2020
Air quality forecast assessment	Fire radiative energy	RAVE	3 km/Hourly	Sep 6–10, 2020
	Physical fields	RAP^g^	3 km/Hourly	Sep 6–10, 2020
	Surface PM2.5 mass concentration	AirNow^h^	–/Hourly	Sep 7–10, 2020

*Note*. Data links are available in the Data Availability section.

Hourly FRP from the Regional Advanced Baseline Imager (ABI) and Visible Infrared Imaging Radiometer Suite (VIIRS) fire Emissions inventory (RAVE; F. Li, Zhang, Kondragunta, et al., 2022) is used as the primary input of fire characteristics (i.e., location and intensity). RAVE provides near‐real‐time hourly FRP over the CONUS based on the ABI sensors onboard the Geostationary Operational Environmental Satellite R Series (GOES‐R), and the VIIRS sensors onboard the Suomi National Polar‐orbiting Partnership (SNPP) and Joint Polar Satellite System (JPSS) satellites. Data flagged as medium and high data quality (quality flag equals to 2 and 3) are used. As a satellite measurement based product, RAVE is regarded as an observation in this study, with the verification of its uncertainties beyond the scope of this work. Although the RAVE data used here is beta version, it should not affect the primary objective of this study that is trying to duplicate near‐real‐time fire products (e.g., RAVE) and provide an estimation of future fire activities.

The HRRR version 3 (HRRRv3; Alexander et al., 2010; Dowell et al., 2022; https://rapidrefresh.noaa.gov/hrrr/) is an operational atmospheric model at NOAA/National Centers for Environmental Prediction (NCEP), providing near‐real‐time hourly weather forecasts for the US. Selected meteorological parameters (T, SH, PRATE, WS and WD) from the HRRRv3 analysis fields at forecast hour F00 are used. Note that the PRATE reported by HRRRv3 is instantaneous rate (kg m^−2^ s^−1^) and is then converted to hourly accumulative rate (kg m^−2^ h^−1^) in this study.

ST and VHI are based on the annual surface type product (AST; C. Huang et al., 2022) and the vegetation health product (VHP; Yang et al., 2018), respectively, from VIIRS. VIIRS AST follows the International Geosphere‐Biosphere Program (IGBP) land cover classification that is commonly used in regional/global scale AQF models. VHI is a combination of the vegetation condition index (VCI) and temperature condition index (TCI), describing the moisture and temperature related vegetation stresses, respectively (Bugalho et al., 2019; Kogan, 1997; Kogan et al., 2021). VHI is typically defined as the average of VCI and TCI. However, previous studies indicated that the vegetation development is mainly restricted by temperature over mid to high latitude regions in the Northern Hemisphere as higher temperatures speed up vegetation growth (Karnieli et al., 2006; Tateishi & Ebata, 2004). Hence, modified VHI is calculated as follows in this study to better represent the general vegetation condition over CONUS:

(1) VHI=0.3×VCI+0.7×TVI

The modified VHI shows consistently negative differences compared to the standard VHI (i.e., the average of VCI and TCI) with an average difference ∼3% annually. Similar regional and seasonal variabilities are found between two definitions (see Appendix 2 in Supporting Information S1). However, the 30‐to‐70 partition used in Equation 1 is a brief assumption without explicit sensitivity tests which may introduce possible uncertainties to the training results.

The climatological FH of 2020 derived from the Global Ecosystem Dynamics Investigation (GEDI) measurements blended with Landsat archive data (Potapov et al., 2021) is used. The integration of ST, VHI and FH is expected to represent the critical fuel characteristics for fire behavior modeling (e.g., fuel type, loading, and flammability). In addition, ELV based on the global product derived by Yamazaki et al. (2017) is obtained from the GriddingMachine database (Wang et al., 2022). Details of each data set can be found in cited references.

For the assessment to air quality forecasting model application, fire radiative energy (FRE) from RAVE and FIRA, which is derived from FRP following F. Li, Zhang, Kondragunta, et al. (2022), and the meteorological fields from the Rapid Refresh (RAP; Benjamin et al., 2016) model during September 6th–10th, 2020 (6th for model spin‐up and 7th–10th for target fire period) are used to drive UFS‐Smoke. RAP is an extended version of HRRR covering a larger domain of North America. The ground‐based observations of PM_2.5_ mass concentration observations from the US Environmental Protection Agency (EPA) AirNow air quality monitoring sites in California during September 7th–10th are used for model evaluation. Because UFS‐Smoke considers fire‐related smoke as the only emission source, it is important to select the sites with hazardous PM_2.5_ levels dominantly affected by the smoke emissions from fire activities. Three criteria are applied for site filtering:

● The daily maximum of AirNow PM_2.5_ observation and UFS‐Smoke surface smoke concentration at a given site should exceed 35 μg m^−3^ according to the National Ambient Air Quality Standards (NAAQS).
● The daily maximum of AirNow PM_2.5_ observation at a given site should exceed the 95th percentile of all the sites statewide.
● The timeseries of UFS‐Smoke surface smoke concentration at a given site should have a positive trend.

The first and second criteria are used to select the sites with extremely high PM_2.5_ concentrations, which are usually associated with smoke emissions during fire season. The third criterion further ensures the selected sites are affected by smoke emissions with continuous incoming smoke plumes. Moreover, picking the polluted sites that are both captured by AirNow observation and UFS‐Smoke simulation can minimize the influence of uncertainties within UFS‐Smoke associated with mechanisms such as smoke transport, which are not the focus of this study. Hence, 33, 21, 14, and 14 sites on September 7th, 8th, 9th, 10th are selected with the daily statewide 95th percentiles as 72, 112, 75, and 117 μg m^−3^, respectively.

Furthermore, to focus on the contribution of fire activities to PM_2.5_ concentrations, background influence from local emissions (e.g., transportation and industry) is removed from AirNow records. Following O'Doherty et al. (2001), the root mean square (*σ*) of PM_2.5_ concentration lower than the median value during the 4‐day period (September 7th–10th) at each site is first calculated. The PM_2.5_ concentrations over 3*σ*, which is site dependent, are identified as “polluted” conditions, otherwise are “background” conditions. The average PM_2.5_ concentration of the background condition is then determined as the “baseline.” Therefore, by assuming the smoke emissions from fire activities are the primary source of high PM_2.5_ concentrations under the polluted condition and the background condition is dominated by local emissions, the contribution of local sources to PM_2.5_ concentration can be quantified and removed from analysis.

### 2.2 Fire Intensity and spRead forecAst (FIRA)

**Figure 1:**
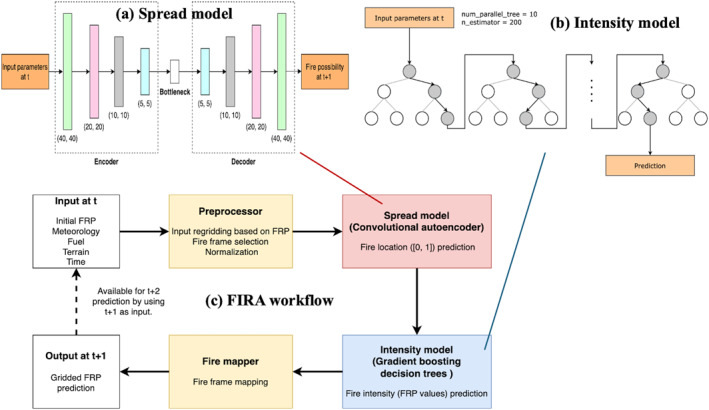
Structures of (a) the spread model, (b) the intensity model, and (c) the forecast workflow in FIRA.

The core of FIRA is two ML models that are used for the predictions of (a) fire spread (i.e., the spatial distribution of FRP; hereafter referred to as the “spread model”), and (b) fire intensity (i.e., FRP values; hereafter referred to as the “intensity model”) for the next time step (e.g. *t* + 1). Since the spread of fire is mainly determined by concurrent fire environment (e.g., weather and fuel), the ML models are designed to capture the real‐time variability of fires and predict for the next timestep only. After the ML models are properly trained, they are further applied to a forecast workflow for multiple forecast time (e.g., *t* + 2, *t* + 3 and so on). The next‐hour‐only feature of the ML models not only reduces the complexity of model design but also provides the flexibility of adjustable forecast time for potential AQF model applications. The structure of the two ML models and the forecast workflow are shown in Figure 1 with details described in this section. Technical code configurations and hyperparameters are provided in Appendix 3 in Supporting Information S1.

#### 2.2.1 ML Model Configuration

##### 2.2.1.1 Fire Spread Prediction Model

The spread model is a convolutional autoencoder (CAE) model, a convolutional neural network (CNN; Yamashita et al., 2018) as the main learning model coupled with autoencoder structure (Shrestha & Mahmood, 2019). With CNN's great capability of learning the two‐dimensional characteristics within data sets, CAE models are often used for medical image processing (M. Chen et al., 2021; Geng et al., 2015; Yildirim et al., 2018) and have been recently used for fire forecasting with good accuracy (Bergado et al., 2021; Cheng et al., 2022). A CAE model (Figure 1a) consists of an encoder that generates compressed representations by downsampling and extracting key features from the input data, a bottleneck where the training processes (a CNN model in this study) take place, and a decoder that reconstruct the trained representations to the original size. Usually, a filtering window (3 × 3 in this study) is given for feature extraction in the encoder. For example, the maximum value within the filtering window will be extracted as the key feature and passed to the next step. The feature extraction process can improve the efficiency of training processes and save computational resources by reducing data noise and dimensionality without losing the important information in the data set. In this study, the spread model is built using the Python Keras and Tensorflow packages (Chollet, 2017).

The spread model is trained by sets of data frames, including the 12 input variables listed in Table 1, with fires at the center based on the initial FRP input. To improve the training efficiency and accuracy, the FRP frames are binarized (i.e., 0 and 1 only; the binary frames) to represent the presence of fire activities as ones and zeros indicate the occurrence and absence of fires, respectively. In addition, the data frames are upscaled and the frame size increases by 8 times to increase frame resolution for better spatial representation. Ideally, the CAE model would treat the given fire frames as images and learn the within two‐dimensional structure (the spatial distribution of fires in this case). The model prediction, which has a range between 0 and 1, shows the possibility of fires reaching a specific grid point. Grids with model predictions equal to or higher than 0.2 (i.e., possibility of 20%) are identified as active fires and therefore given a binary digit as 1, otherwise are identified as no fires and given a binary digit as 0. This threshold (model predictions equal to 0.2) is an experimental value based on a simple sensitivity test by examining the model performance with different thresholds values (see Appendix 4 in Supporting Information S1 for details). The re‐classified binary frame prediction, downscaled to the original frame size, will be the output of the spread model.

##### 2.2.1.2 Fire Intensity Prediction Model

The intensity model is an optimized gradient boosting decision trees (GBDT) model for FRP value prediction based on the Python XGBoost package (T. Chen & Guestrin, 2016). GBDT, which is well‐known for its training efficiency and accuracy in solving regression problems, is a decision trees (Quinlan, 1986; Song & Lu, 2015) based algorithm while multiple decision trees are trained sequentially to prevent overfitting and improve prediction accuracy (Figure 1b). Each decision tree minimizes the errors from the previous decision tree, achieving the final GBDT model with an improved accuracy eventually.

The intensity model is trained by the input variables at the grid points with active fires (i.e., binary digit equals 1 in the binary frames) while original FRP values are used. Following RAVE, valid fires are represented by valid non‐zero FRP values, otherwise are zeros. Unlike the binarized output from the spread model, the intensity model generates predictions with realistic FRP values. Prediction with values less than 1 MW will be treated as extinguished fires and assigned as 0 MW in the output. The modified FRP frame prediction will be the output of the intensity model.

##### 2.2.1.3 Data Preprocessing and Model Training

All variables are first regridded to share the same spatial coordinate as the initial FRP data, namely RAVE in this study. For a given grid point in the RAVE coordinate, ST and HOUR are determined by the values from the nearest grid point in the original coordinate while other input variables are determined through spatial linear interpolation. Afterward, sets of data frames are identified based on the initial FRP data, and the binary frames are further generated for the spread model. Each data frame covers a square domain of 15 × 15 km^2^ (i.e., 5 × 5 grid). Fires are assumed to spread within this area regardless of the spread directions while the spread rate of fire usually falls in a range of 1–10 km hr^−1^ (Liu et al., 2021). Sets that contain data frames with missing values are abandoned (∼20%–30% of the total data) as valid values are required by ML algorithms in the training process. In addition, sets associated with isolated weak fires, which contain only one non‐zero FRP data point at the center of the data frame with values less than 15 MW, are assumed to be contained/dying fires with no spreading and are removed from the data set. This threshold is defined based on an analysis of 5‐year FRP records from the Moderate Resolution Imaging Spectroradiometer (MODIS) satellite documented by Giglio et al. (2006), in which a low annual FRP value around 15 MW is reported for extensive cropland fires. The 15 MW value is adopted as the minimum FRP for actively growing fires and fires less than 15 MW are assumed to be contained with no future spreading in this study. Following steps are applied to the spread model only. Specific input variables are transformed to Gaussian distribution by taking square roots on SH, taking log values on FRP and PR, and taking cubic roots on FH and ELV. All variables undergo max‐min normalization as required by ML algorithms and for better learning efficiency.

Sets of data frames associated with fires in August are used as test data for model evaluation, and the rest are categorized into three groups based on the seasonality of fire activities: winter (January, February, November, and December), spring (March, April, and May), summer/fall (June, July, September, and October). Each group is further separated into daytime and nighttime subgroups to account for the diurnal variability of fire behaviors (six subgroups in total). Lastly, 50% of the data is randomly selected from each subgroup for training and the rest 50% is used for validation. This is critical for ML models to ensure both the training and validation data encompass the fire characteristics of fire activities across different times and seasons. Therefore, about 185,000, 180,000, and 45,000 sets of data frames are used for model training, validation, and evaluation, respectively. Because of the big amount of the training and validation data, cross validation is not performed.

#### 2.2.2 Forecast Workflow

The forecast workflow of FIRA consists of a data preprocessor, the spread model, the intensity model, and a fire mapper (Figure 1c). First, the data preprocessor takes the gridded input data (e.g., FRP, meteorological parameters and fuel parameters) and generates inputs for the ML models by following the preprocess steps described in Section 2.2.1. Although the geographic information (latitude and longitude) is not used in the ML models, it is necessary for the identification of the geographic location of fire predictions when generating the final gridded product for future applications.

Afterward, the preprocessed data frames are passed to the spread model for fire spread prediction. Based on the outputs from the spread model (i.e., binary frame predictions), grid points with active fires (fire grids) can be identified and corresponding input variables at the fire grids are collected. By passing these input variables to the intensity model, the FRP predictions at identified fire grids can then be generated. Finally, the outputs from the intensity model (i.e., FRP frame predictions) are passed to the fire mapper, where the FRP frame predictions will be re‐mapped to regular latitude/longitude coordinates based on the geographic information from the preprocessor, and the gridded FRP prediction for the next time step will be available as the final product. By default, the final product shares the same data format and spatial coordinates as the initial FRP input for easy application and implementation to AQF model systems.

The major advantage of this workflow is its flexibility of forecast time by feeding the gridded product into forecast cycles. For instance, input data at initial time *t* is used for the FRP predictions at time *t* + 1, which can then be passed to the next forecast cycle along with other input variables (e.g., T and SH) at time *t* + 1 and generate the FRP predictions at time *t* + 2. By repeating this process, FRP predictions for multiple forecast time steps will be available. In this study, the ML models are trained by the hourly data from RAVE and the workflow generates FRP predictions on an hourly basis.

### 2.3 Experimental UFS Smoke Forecast Model (UFS‐Smoke)

The Unified Forecast System coupled with smoke (UFS‐Smoke; Ahmadov et al., 2022) model is an experimental model based on the Unified Forecast System (UFS; Alexander & Carley, 2021) developed at NOAA, incorporating three‐dimensional transport, mixing, and dry and wet deposition of smoke emitted from wildland fires. Smoke is treated as a chemically inert tracer, and no other emission sources are included. UFS‐Smoke covers the CONUS domain at a 3 km resolution and has 65 vertical levels. The meteorological initial and boundary conditions are from NOAA's continental‐scale, hourly updated RAP at a 13 km resolution.

Smoke emissions are estimated using the hourly FRP reported by RAVE. Fire size/intensity (i.e., the energy released from fires) is determined by the FRP data and used to calculate plume injection heights for the flaming emissions following a modified version of Freitas et al. (2007, 2010). Two emission modes are implemented in UFS‐Smoke. In the forecast mode, daily FRP from the prior day is assumed to be the total fire size for the target day, and hourly smoke emissions are calculated based on the prediction of Hourly Wildfire Potential index (HWP; James et al., 2023). Persistence in location is assumed with no fire spreading included. In the retro mode, near‐real‐time hourly FRP from the target day is directly used for emission estimation. Technically, the retro mode should better represent fire activities in reality compared with the forecast mode. The UFS‐Smoke model is still experimental and undergoing intensive development and testing at NOAA GSL and one of the research versions of UFS‐Smoke model is used in this study. A full description of the UFS‐Smoke model will be presented in a separate publication in the future.

In this study, UFS‐Smoke simulations are conducted over the CONUS domain for a 4‐day period during a severe California wildfire event on September 7th–10th, 2020 with 1 day spin‐up. Results based on various FRP inputs, including RAVE FRP and FIRA FRP predictions, are analyzed for the assessment of FIRA FRP for potential AQF applications. FIRA forecast workflow is initialized 1 hr prior to each selected day and a 24 hr forecast is generated. For instance, the workflow is initialized at 2300 UTC on September 6th and FRP forecast for the following 24 hr is generated to represent the fire activities during 0000–2300 UTC on September 7th. FIRA FRP predictions are converted to FRE following F. Li, Zhang, Kondragunta, et al. (2022) and formatted to RAVE‐like data format before applying to UFS‐Smoke.

### 2.4 Statistical Analysis

Table 2 summarizes the statistical scores/matrices used in this study. The prediction of fire spread (i.e., spatial distribution of FRP) is evaluated by frame similarity (FS), which describes the level of similarity between two data frames. For the evaluation of the prediction of fire intensity (i.e., the FRP values) generated by the intensity model, three additional statistical scores are used including mean bias (MB), coefficient of determination (*R* ^2^), and root mean square error (RMSE). In addition, to evaluate the accuracy of gridded FRP prediction generated by the fire mapper, the alarm rates of true alarms, false alarms and miss alarms are calculated as the measures of good forecast, false recognition, and missing case, respectively. The three statistic scores described above (i.e., MB, *R* ^2^, and RMSE) and the variances of PM_2.5_ observation from selected AirNow monitoring sites and surface smoke concentration from four UFS‐Smoke simulations are analyzed for the assessment to AQF model application. Furthermore, to understand the relationships between input variables and predictions from the ML models (i.e., the fire spread and FRP predictions), the permutation variable importances (VIs; McGovern et al., 2019) of 12 input variables are estimated for the spread and intensity models. Detailed definitions and formulations of these statistical scores and VI can be found in Appendix 5 in Supporting Information S1.

**Table 2:** Statistical Scores Used for Model Evaluation, Application Assessment, and the Analysis of Variable Contributions to the ML Models

Application	Statistical score
Evaluation of fire spread prediction	Frame similarity (FS)
Evaluation of fire intensity prediction	Mean bias (MB), Coefficient of determination (*R* ^2^), Root mean square error (RMSE)
Evaluation of gridded FRP prediction	True/False/Miss alarm rate
Verification of the contribution of input variables in the ML models	Variable importance
Comparison of UFS‐Smoke results and AirNow observations	Mean bias (MB), Coefficient of determination (*R* ^2^), Root mean square error (RMSE), Variance

## 3 Fire Prediction Evaluation

### 3.1 Model Evaluation

The two ML models in FIRA are evaluated against the satellite‐based FRP data from RAVE. To understand the influence of fire behaviors on model performance, fires in test data are categorized into two types based on fire intensity. According to the seasonality of 5‐year FRP records from MODIS (Giglio et al., 2006), fires with the maximum FRP values exceeding 80 MW that are often associated with forests in the western US are identified as wildfires, otherwise are classified as agricultural/grassland fires which usually are prescribed burns in the central and southeastern US.

**Figure 2:**
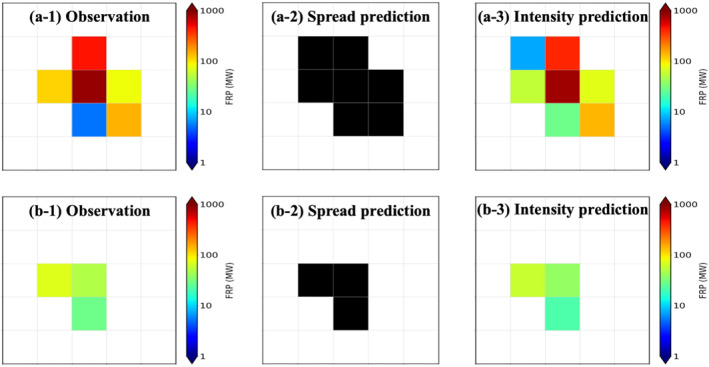
Comparison of the observed RAVE FRP and the predictions generated by the spread model and the intensity model for selected (a) wildfire and (b) agricultural/grassland fire cases. Note that the transparent grids are referring to FRP values equal to 0 MW (i.e., grids without fires) instead of missing values for better visualization.

Figure 2 shows the comparison of the FRP frames from RAVE, binary frame predictions generated by the spread model, and FRP frame predictions generated by the intensity model for selected examples of a wildfire case and an agricultural/grassland fire case during the test period (August 2020). These cases are randomly selected while agricultural/grassland fires with only single FRP point at the center in the observation (i.e., no spreading) are skipped. Table 3 shows the statistical analysis of the predictions from spread and intensity models compared to RAVE FRP for all fires, wildfires and agricultural/grassland fires during the test period. Overall, the ML models capture fire spread with FS about 94% for both the spread and intensity models. The FRP predictions from the intensity model also show good agreement with RAVE FRP with the average MB and *R* ^2^ of −6.15 ± 41.65 MW and 0.71 ± 0.36, respectively. The negative MB indicates that the intensity model generally underestimates FRP values, probably due to the massive number of agricultural/grassland fires with relatively low FRP in the training data. However, the average RMSE (56.40 ± 131.46 MW) is relatively high since it describes about 20% of the average FRP, indicating possible uncertainties in FRP predictions.

**Table 3:** Statistical Results of the Predictions From Spread and Intensity Models Compared to RAVE FRP for All Fires, Wildfires and Agricultural/Grassland Fires During August 2020

	All	Wildfire	Agricultural/Grassland
Average RAVE FRP (MW)	274.48 ± 473.46	330.63 ± 508.25	28.74 ± 22.44
FS–Spread model (%)	94.13 ± 7.73	91.86 ± 9.26	96.46 ± 4.73
FS–Intensity model (%)	94.57 ± 6.94	92.55 ± 8.22	96.63 ± 4.45
MB (MW)	−6.15 ± 41.65	−14.34 ± 56.70	2.24 ± 8.95
R^2^	0.71 ± 0.36	0.64 ± 0.36	0.82 ± 0.31
RMSE (MW)	56.40 ± 131.46	100.28 ± 171.09	11.44 ± 31.71

The difference in the performance of fire spread prediction between two fire types is minor, while the average FS for wildfires and agricultural/grassland fires are about 92% and 96%, respectively. However, the ML models tend to underestimate (overestimate) the fire intensity for wildfires (agricultural/grassland fires) with a negative (positive) MB about −14 MW (2 MW). The over and under estimations are associated with the limitation of ML algorithms. The number of agricultural/grassland fires is about double of the number of wildfires in the training data. Because the training data is not categorized into different fire types in the training process, the ML models are capturing the common behavior of all fires and thus producing relatively higher (lower) values for low (high) FRP cases. In addition, satellite fire detection usually is less reliable for low‐intensity fires due to cloud screening and minor differences in brightness temperature between fires and surrounding regions (F. Li et al., 2020), leading to potential uncertainties in the representation of agricultural/grassland fires in the intensity model. The limited‐varying FRP values of agricultural/grassland fires (1–79 MW by definition) may also contribute to relatively low absolute MBs and RMSEs compared to wildfires (80–∞ MW by definition) when fires are categorized in the analysis stage. The examples in Figure 2 also show similar results, while the spread prediction introduces one grid of difference from RAVE FRP and the FRP predictions have larger biases for the wildfire case (Figure 2a) compared to the agricultural/grassland fire case (Figure 2b). In addition, the average RMSEs (100.28 ± 171.09 MW for wildfires and 11.44 ± 31.71 MW for agricultural/grassland fires) describe about over 30%, even near 40% for agricultural/grassland fires, of the average FRP values. This could lead to considerable concerns in the forecast for a longer period as model biases may propagate with forecast time, which are discussed in Section 3.3.

**Figure 3:**
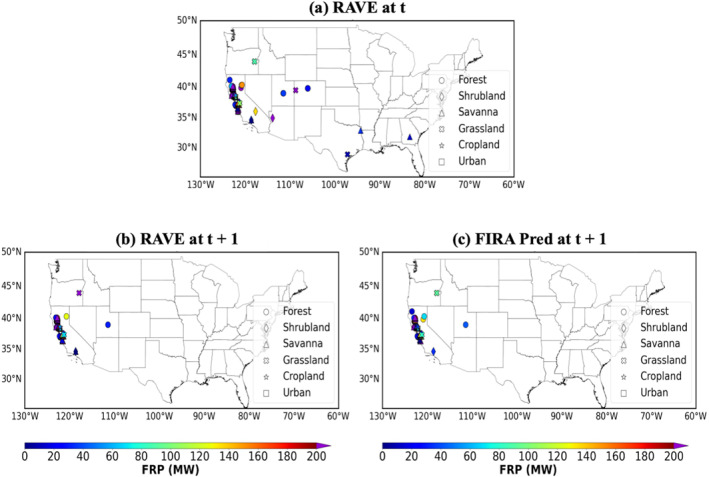
RAVE FRP at (a) initial time t and (b) forecast time *t* + 1, and (c) remapped FIRA FRP prediction at forecast time *t* + 1 for selected cases initializing at 1200 UTC on 20 August 2020.

**Figure 4:**
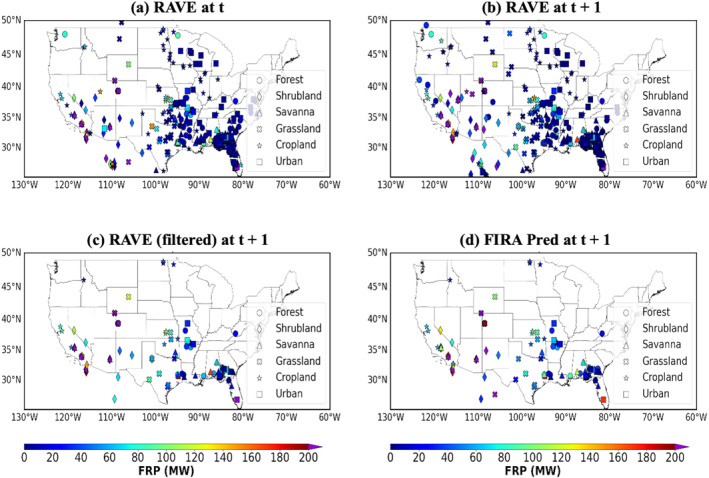
Same as Figure 3 but initialing at 1800 UTC on 4 August 2020. Note (c) is the RAVE FRP at forecast time *t* + 1 shown in (b) without isolated weak fires.

Figures 3 and 4 illustrate two examples of the results after mapping the FRP frames back to gridded CONUS domain, and Table 4 shows the alarm rates associated with different STs for the selected cases. The first case is initialized at 1200 UTC on 20 August 2020, when forest wildfires over the western US were dominated (Figure 3). The ML models capture the trend of fire spread in general with true alarm rates of 60%–70% for all fires and the primary forest, shrubland, and savanna fires in California. However, moderate miss alarm rates of about 10%–20% are found, probably due to the selection of fire frames based on an assumption of maximum spread rate of fire in the ML model configuration. Certain wildfires with high spread rates may grow beyond the defined domain, resulting in missing fires in model predictions (see Section 3.3 for more discussions). The ML models also successfully capture the dying fires in Utah, Colorado and Texas.

**Table 4:** Alarm Rate (%) of FIRA FRP Predictions With Different Surface Types for the Selected Cases Shown in Figure 3

	All	Forest	Shrubland	Savanna	Grassland	Cropland	Urban
Initial time: 20200820 1200 UTC							
True alarm	68.61	63.41	58.33	71.79	66.67	–	–
False alarm	18.98	19.51	33.33	16.67	33.33	–	–
Miss alarm	12.41	17.07	8.33	11.54	0.00	–	–
Initial time: 20200804 1800 UTC							
All fires							
True alarm	32.54	25.00	53.33	36.08	41.54	18.82	23.26
False alarm	3.33	3.13	6.67	3.09	3.08	1.17	2.33
Miss alarm	64.13	71.88	40.00	60.82	55.38	80.00	74.42
Isolated weak fires filtered							
True alarm	75.69	69.57	76.19	76.09	75.00	76.19	90.91
False alarm	7.73	8.70	9.52	6.52	5.56	4.76	9.09
Miss alarm	16.57	21.74	14.29	17.39	19.44	19.05	0.00

*Note*. No cropland and urban fires are found in the 20200820 case. Alarm rates compared with the simulations with all fires and isolated weak fires filtered are calculated for the 20200804 case.

The second case, initializing at 1800 UTC on 4 August 2020, was dominated by the massive savanna, grassland and cropland fires in the central and southeast US (Figure 4). The ML models fail to predict the fire propagation with miss alarm rates up to 60% and even 80%. In the data preprocessing stage, weak fires with FRP values less than 15 MW are identified as dying fires and are assumed to be isolated with no spreading. Also, most cropland fires are prescribed fires and usually only last for a couple of hours. Hence, these cropland fires may be treated as dying fires and do not propagate in the ML models. In fact, the performance of the ML models significantly improves with true and miss alarm rates around 70% and 20% for all STs, respectively, when comparing with the RAVE FRP with weak fires filtered (Figure 4c). These results indicate possible uncertainties associated with the assumption and removal of dying fires in data preprocessing and model configuration.

### 3.2 Variable Importance Analysis

**Figure 5:**
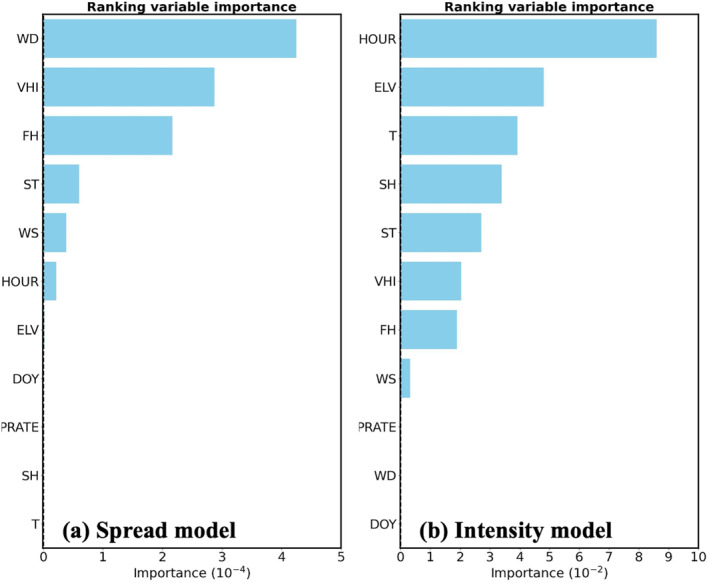
Ranking variable importance of the training parameters except for FRP used in the (a) spread model and (b) intensity model. The VIs for FRP for both ML models are over 0.9, which are far beyond the magnitude of other parameters and thus are not presented here. Note that since the scales of the model outputs from the two ML models (the spread model predicts the possibility of getting fires while the intensity model predicts the FRP value), the scales of VI could vary. Ranking VI shows the relative contribution of variables in the ML models and the scales should not affect the results.

Figure 5 shows the ranking VI for the training parameters, except for FRP, used in the two ML models, representing the relative contribution of each input variable to model predictions. The VIs for FRP for both ML models are far higher than other parameters and thus are not shown in the figures. For the spread model, FRP plays a dominant role in spread prediction with the highest VI (>0.9) by providing the initial fire locations to the model that largely affect the fire propagation in the future time steps. Among non‐FRP variables, the considerable contribution from wind components (WD and WS) is expected as the spread of fires is highly correlated with horizontal winds according to previous field measurements and laboratory experiments (Beer, 1993; Cheney & Gould, 1995; Cheney et al., 1993, 1998). WD shows a relatively high VI compared to other parameters besides FRP since it determines the forward direction of fires, that is the key feature learned by the spread model. Fuel parameters (VHI, FH, and ST) also show good contributions to spread prediction by providing critical fuel characteristics to the spread model (Cheney et al., 1993; Rothermel, 1972). In addition, the near‐zero VIs for ELV, DOY, PRATE, SH, and T are because of the relatively low variability (i.e., the ratio of standard deviation to mean less than 0.01) in the 15 × 15 km^2^ area within selected data frames compared to other parameters, making the spread model take less advantage of these parameters.

Similar to the spread model, FRP plays a dominant role (VI > 0.9) in the intensity model since it provides the initial fire characteristics (e.g., fire intensity and burning stage) to the model. The significant contribution from HOUR is associated with the diurnal cycle of fires while fires are more intense during daytime (Andela et al., 2015; Giglio, 2007; F. Li et al., 2019). However, it is worth nothing that using HOUR as the indicator of fire diurnal cycle may introduce potential uncertainties in particular for fire activities with no distinct diurnal variability. Following HOUR, meteorological parameters (T, SH, and WS) and fuel parameters (ST, VHI, and FH) show considerable contributions in the intensity model, reflecting the impacts of weather conditions and fuel characteristics on FRP values, respectively. These results are consistent with previous studies in that weather conditions and fuel characteristics play critical roles in fire severity (Jain et al., 2020). However, although many studies reported the significant contribution of precipitation, PRATE shows a near‐zero VI here. It is because only selected fires are used in this study. More than 99% of the PRATE data is below 0.1 kg m^−2^ h^−1^ (Appendix 1 in Supporting Information S1) and is relatively consistent during burning periods, introducing nearly no variabilities in the intensity model. Since precipitation mitigates fire severity (Jain et al., 2017), missing its variability could introduce potential uncertainties to the intensity model. Also, PRATE represents the instantaneous precipitation at specific time, while fire behaviors are mostly affected by accumulated precipitation through a period of time. In addition, ELV affects fire intensity by partially determining the fire types and ambient environment (Liu et al., 2021). Most fires often occur in mountainous regions with an average ELV around 520 m, in particular for the intense fires such as forest and shrubland fires with an average ELV about 630 m. WD shows nearly no contribution to FRP values since it mainly affects fire spread rather than fire intensity.

To better verify the effects of non‐FRP parameters, additional ML models are trained based on the same model configurations described in Section 2 but with different input variables, and VI analysis are performed (Appendix 6 in Supporting Information S1). Although non‐FRP variables (meteorological conditions and fuel characteristics) show low VIs, removing them from the training data leads to an increased average RMSE by ∼3%, showing that non‐FRP variables still affect model performance to some extent despite the low VIs. In addition, fuel and fire type related parameters (e.g., VHI, ST, and ELV) become the dominant contributors when removing FRP from the training data, indicating that the ML models are mainly predicting the flammability of vegetations/fuels with weak representation of the physical processes and meteorological drivers of fire activities. This is not surprising as the spread model is designed to learn the possibility of fires reaching a specific grid point, although a better presentation of the physical processes of fire spread is expected.

### 3.3 Multi‐Hour Forecast

Two fire cases in 2020 and the Park Fire in 2024 are selected for the evaluations of the multi‐hour forecast generated by FIRA forecast workflow described in Section 2.2.2. The initial times are determined based on preparatory analysis of the fire activities during fire periods. While the ML models are trained and validated based on the data in 2020, it is important to examine model's generalizability for different periods. Since the ML models are designed for the prediction of existing fires, the fires in the next hour outside the selected 15 × 15 km^2^ domain identified in the data preprocessor are regarded as new ignitions and removed from analysis. Isolated weak fires are also removed for better comparison.

#### 3.3.1 California Wildfire on 20 August 2020

**Figure 6:**
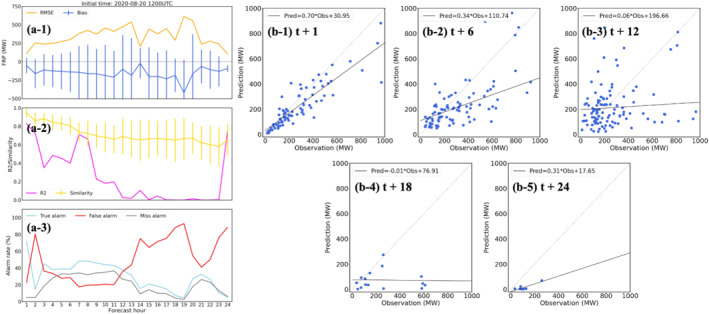
Evaluation of the 24 hr forecast for the California wildfires on 20 August 2020, including (a) the timeseries of statistical results (i.e., RMSE, MB, *R* ^2^, FS, and alarm rates) and (b) the comparison of paired observations (i.e., RAVE FRP) and FIRA FRP predictions at multiple forecast time 1, 6, 12, 18, and 24 hr.

The first case is the California wildfires on 20 August 2020, which were mainly forest fires with the average and maximum FRP values about 350 MW and over 6,000 MW, respectively. The workflow is initialized at 1200 UTC on September 20th and a 24 hr forecast (forecast time = 1–24 hr) is generated. Figure 6a is the hourly timeseries of statistical scores (MB, RMSE, FS, *R* ^2^, and alarm rates) of FIRA predictions compared to RAVE data. Results show that FIRA workflow introduces an increasing trend in RMSE from 250 MW to over 500 MW along with the forecast period, and consistently underestimates FRP values by about 200 MW (Figure 6a‐1). The negative MBs are expected based on the results found in model evaluation (Table 3), while model errors accumulate with forecast time leading to increasing RMSEs. Moreover, *R* ^2^ values (Figures 6a‐2) significantly decrease after 6 hr and even drop to near zero after 12 hr. This trend can also be found in the scattering plots shown in Figure 6b, while the correlations between the RAVE FRP and FIRA FRP predictions significantly degrade and FIRA FRP predictions are much lower than RAVE FRP after 12 hr forecast (Figures 5, 6).

**Figure 7:**
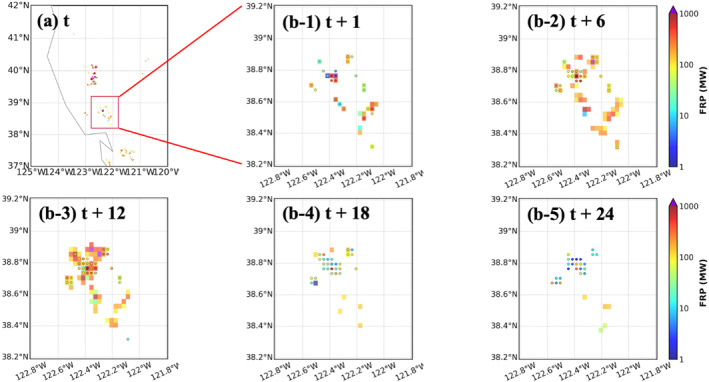
Spatiotemporal variation of RAVE FRP (shaded) at (a) initial time, and RAVE FEP covered by FIRA FRP (dots) at (b) forecast time 1, 6, 12, 18, and 24 hr for the California wildfires on 20 August 2020. The red box in (a) demonstrates the location of the selected fire incident.

In contrast, FIRA workflow shows an overall good performance in fire spread prediction with FS over 60% (Figure 6a‐2) even after 24 hr (i.e. forecast time = 24 hr). The spatial distribution of gridded FRP also shows that FIRA workflow successfully captures the majority of wildfires in terms of geographic locations (Figure 7). Although the true alarm rate consistently drops after the 3 hr forecast (Figure 6a‐3), it keeps a relatively consistent value ∼40% (∼20%) for the first 12 hr (later 12 hr), indicating a stable performance in multi‐hour fire spread predictions. The alarm rates show diurnal variabilities as the ML models tend to have more true and miss alarms during daytime and increasing false alarms during nighttime. The daytime missing fires are probably due to the limited spread domain defined in the spread model, as discussed in Section 3.1, as well as the more intense fire activities and spreading in daytime (Andela et al., 2015; F. Li et al., 2019). On the other hand, the high false alarm rates indicate that the ML models do not learn the diurnal variability of fire spread properly and thus predict overactive fire activities during nighttime compared to RAVE. The high false alarm rates are likely due to the fact that most fires happened in the daytime (Appendix 1 in Supporting Information S1) and the limited number of nighttime fires in the training data is not sufficient for the ML models to learn. This is consistent with the results of variable importance analysis (Figure 5a) where HOUR shows relatively low contribution in the spread model. Missing the physical connections between fire behaviors and meteorological parameters (e.g., T and SH), which usually have distinct diurnal patterns, could also prevent the ML models from identifying daytime and nighttime fire activities. It is worth noting that the high false alarm rates at forecast time 2 hr is because of the large amount of missing FRP data in RAVE. Given the active fire activities at forecast time 3 hr, the missing data should not be associated with dying fires but the uncertainties from satellite measurements such as clouds.

#### 3.3.2 Agricultural Fire on 4 August 2020

**Figure 8:**
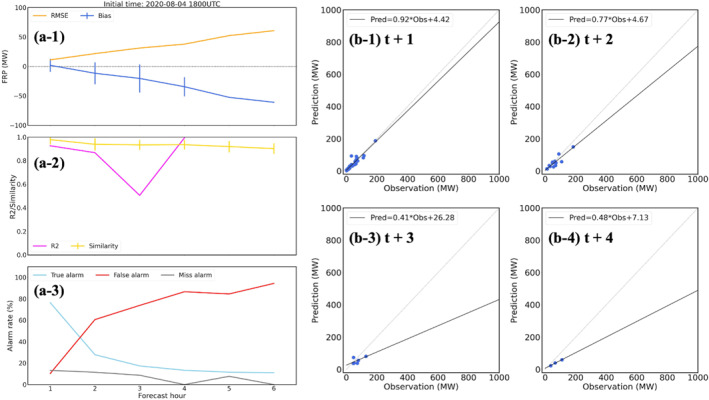
Same as Figure 6 but for the agricultural fires in the southeastern US on 4 August 2020.

**Figure 9:**
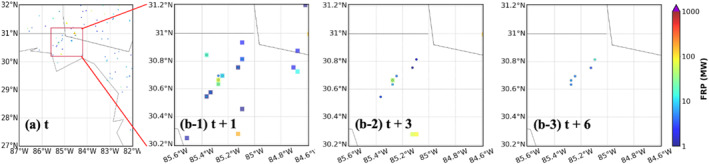
Same as Figure 7 but for the agricultural fires in the southeastern US on 4 August 2020.

The second case is the agricultural fires in the southeastern US on 4 August 2020 with an average FRP around 18 MW. The workflow is initialized at 1800 UTC on August 4th and a 6 hr forecast (forecast time = 1–6 hr) is generated. A shorter forecast period is conducted since agriculture fires usually are prescribed, well‐controlled burns and only last a couple hours compared to wildfires. Figure 8 shows the hourly timeseries of statistical scores (MB, RMSE, FS, *R* ^2^, and alarm rates) of FIRA predictions compared to RAVE data. Since there are less than three pairs of FRP points in FIRA predictions and RAVE data, *R* ^2^s are not calculated for forecast time 5 and 6 hr. Compared to the California wildfire event, lower MBs and RMSEs are found corresponding to lower FRP values. Negative MBs are also found, indicating a general underestimation in FRP values. The high FS (∼90%) shows the good capability of spread predictions when comparing paired fire frames from the ML models and RAVE. The high FS could also be caused by the relatively low resolution (3 km) compared to the scale of the selected fires. However, when comparing the gridded FRP products, FIRA workflow tends to predict overactive fires with decreasing true alarm rates to 20% and increasing false alarm rates to over 60% after 2 hr. According to the spatial distribution of FIRA FRP predictions and RAVE FRP (Figure 9), FIRA workflow seems to capture the dying trend of fires, which is consistent with the results of ML model evaluation discussed in Section 3.1, but with a slower speed. Most fires are extinguished in RAVE at forecast time 6 hr, while FIRA workflow still predicts weak fires over the major burning region with an average FRP about 7 MW (Figures 9a‐3 and 9b‐3). All fires are extinguished after 10 hr in FIRA prediction (not shown), indicating that the ML models successfully capture the trends but fail to predict the correct extinguish time. It may be associated with the relatively long burning periods of wildfires (usually in days, even weeks). Because all fires are equally applied in the training process, long burning periods of wildfires may increase the overall burning periods of non‐wildfire events. These results bring up the difficulties and uncertainties of predicting the multi‐hour fire propagation of weak fires such as agricultural fires in the ML models.

#### 3.3.3 California Wildfire on 26 July 2024

**Figure 10:**
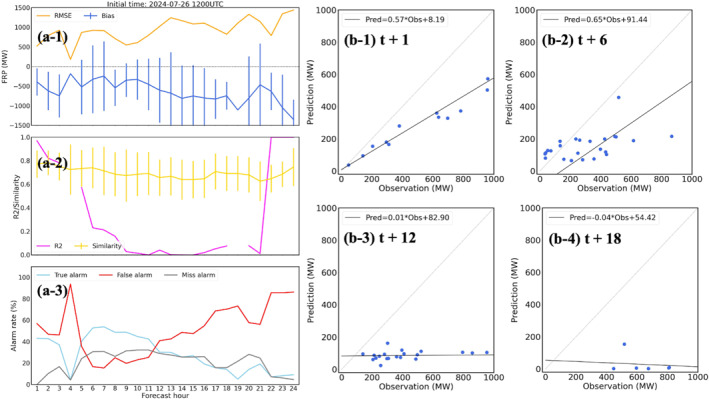
Same as Figure 6 but for the California Park Fires on 26 July 2024.

**Figure 11:**
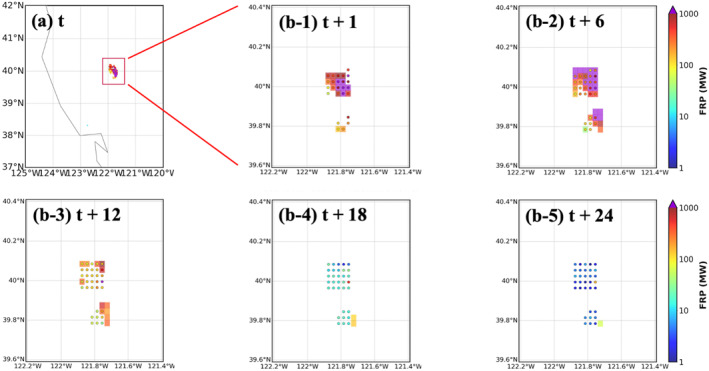
Same as Figure 7 but for the California Park Fires on 26 July 2024.

The third case is the Park Fire, an extremely severe wildfire in Northern California. It ignited on 24 July 2024, and aggressively expended in late July (https://www.fire.ca.gov/incidents/2024/7/24/park‐fire). The workflow is initialized at 1200 UTC on 26 July 2024, with the average and maximum FRP values about 1,070 MW and 10,280 MW on this day, respectively. A 24 hr forecast (forecast time = 1–24 hr) is generated. Figure 11 shows the hourly timeseries of statistical scores (MB, RMSE, FS, *R* ^2^, and alarm rates) of FIRA predictions compared to RAVE data. *R* ^2^ is not calculated and the scatter plot is not shown for forecast time 4 and 24 hr due to limited number of pairs of FRP points in FIRA predictions and RAVE data. Compared to the first case, which is also a California wildfire but in 2020, the FIRA workflow generates higher RMSE from 500 MW to near 1,500 MW for the Park Fire while fire intensities are significantly underestimated. It is due to the incredibly high FRP values far beyond the average FRP values (∼180 MW) used in the training data and there are only around 2.5% of the fires in the training data associated with high FRP over 1,000 MW. However, FIRA FRP predictions and RAVE FRP are significantly correlated with *R* ^2^ over 0.8 at forecast time 1–3 hr (Figures 10a‐2 and 10b‐1), showing the reliable performance of the ML models. The decreasing correlations with forecast time are expected due to accumulated model errors as discussed previously. As for fire spread prediction, FIRA workflow captures the location of fires with consistent FS over 80% when comparing paired fire frames from the ML models and RAVE throughout the entire forecast period. Although overactive fires are predicted with increasing false alarm rates (Figure 10a‐3), the decreasing FRP values represent the dying trend of fires within the fire frame (Figure 11b). Overall, FIRA workflow performs similar performance for the selected two California wildfires (i.e., the first case in 2020 and the Park Fire in 2024), indicating the ML models' capability of generating reliable predictions of fire propagation for various fire periods/seasons.

However, it is important to mention that this comparison is based on the allocated fire frames and new fires (i.e., fires outside the selected fire frames) are removed from analysis. In fact, the Park Fire expended rapidly on 26 July 2024, moved far beyond the assumed 15 × 15 km^2^ spread area, and thus could not be fully captured by the selected fire frames in the ML models (see Appendix 7 in Supporting Information S1 for details). This finding emphasizes the limitations and possible uncertainties associated with the assumption of maximum spread rate of fire and restricted spread area in the ML models as mentioned in Section 3.1.

## 4 Assessment to Air Quality Forecast Model Application

To understand the impacts of FIRA FRP predictions on AQF application, four UFS‐Smoke simulations during a historical California wildfire season (September 7th–10th, 2020) are analyzed in the following sections. All simulations share the same model configurations except for the model mode and source of FRP input, summarized in Table 5. The Base case is based on the near‐real‐time RAVE FRP in retro mode and should be the closest to the reality among four designed simulations. The Fcst case uses RAVE FRP data from the previous day following a forecasting manner (e.g., use FRP from September 6th for September 7th), since measurements for the target day are usually unavailable when generating forecasts. Also, fire spread is not considered in the forecast mode in UFS‐Smoke. Both the Pred and Pred2 cases are based on FIRA FRP predictions, while the FRP values are scaled by a factor of 2 in the Pred2 case as an example of possible treatment for the underestimation issues found in model evaluation. In this section, the Base case is regarded as the “target” for result comparison. Since the major objective of this study is to update the static fires by providing more dynamic fire information to AQF models, the evaluations of UFS‐Smoke and the Base case are beyond the scope of this work.

**Table 5:** UFS‐Smoke Simulations Used for Air Quality Forecast Modeling Application Assessment

Simulation ID	Model mode	FRP input
Base	Retro mode	RAVE FRP
Fcst	Forecast mode	RAVE FRP
Pred	Retro mode	FIRA FRP
Pred2	Retro mode	FIRA FRP x 2

*Note*. Four simulations share the same model configurations except for the model mode (retro or forecast) and source of FRP input (RAVE or FIRA).

### 4.1 Comparison of UFS‐Smoke Simulations

**Figure 12:**
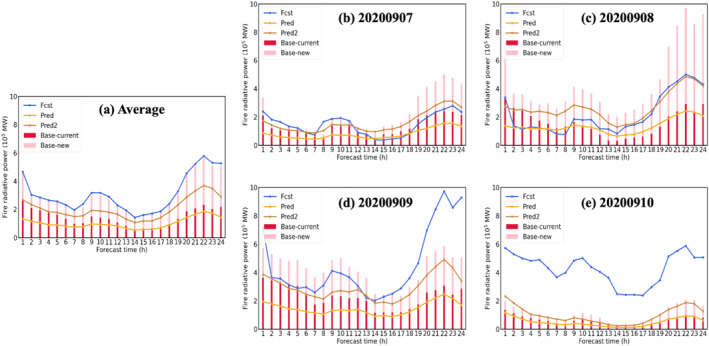
The (a) average 24 hr timeseries of total fire intensity (sum of FRP values) during the selected 4‐day period September 7th–10th, and the 24 hr timeseries of total fire intensity on (b) September 7th, (c) September 8th, (d) September 9th, and (e) September 10th. Red and pink bars represent the total fire intensity associated with current fires and new ignitions for the Base case. Blue, orange and brown lines are the total fire intensity for the Fcst, Pred and Pred2 cases, respectively.

**Figure 13:**
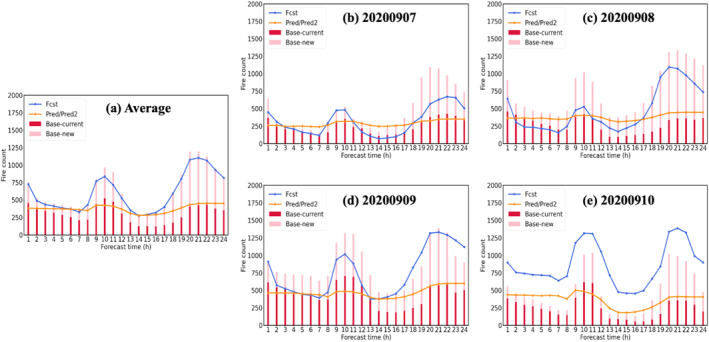
The (a) average 24 hr timeseries of total fire count (number of valid FRP data) during the selected 4‐day period September 7th–10th, and the 24 hr timeseries of total fire count on (b) September 7th, (c) September 8th, (d) September 9th, and (e) September 10th. Red and pink bars represent the total fire counts associated with current fires and new ignitions for the Base case. Blue and orange lines are the total fire intensity for the Fcst and Pred/Pred2 cases, respectively.

In this section, the Fcst, Pred and Pred2 cases are evaluated against the Base case. Figures 12 and 13 illustrate the 24‐hr timeseries of the FRP data used in the four UFS‐Smoke simulations on September 7th–10th, along with the 24‐hr timeseries averaging over the 4‐day period. Similar to the multi‐hour workflow evaluation (Section 3.3), fires in the next hour within the selected 15 × 15 km^2^ domain at current time step are classified as current fires (shown as red bars in Figures 12 and 13), otherwise are new ignitions (shown as pink bars in Figures 12 and 13).

According to the 24 hr timeseries of total fire intensity (i.e., sum of FRP values) shown in Figure 12, on average, RAVE FRP used in the Fcst case align well with the total fires in the Base case, while FIRA FRP predictions in the Pred and Pred2 cases follow the trend of current fires in general and therefore are lower than the total FRP from all fires (current fires + new ignitions) (Figure 12a). This result is expected as FIRA is designed to capture the evolution of existing fires. However, the FRP values in the Fcst case are significantly larger than the Base case on September 9th and 10th, since they are actually based on the FRP data from the previous day (i.e., September 8th and 9th, respectively). For rapid‐varying fires with significant changes in total FRP on September 9th and 10th for instance, using FRP data from the previous days may lead to serious concerns in the representation of fire activities. In contrast, FIRA FRP predictions used in the Pred and Pred2 cases are generally lower than the total FRP in the Base case, and they can better represent the day‐to‐day fire variability especially for current fires, compared with the Fcst case.

Similar situations are also found in the 24‐hr timeseries of fire counts (i.e., number of valid fire grids) shown in Figure 13. The fire counts based on previous‐day RAVE FRP data used in the Fcst case agree well with the Base case in general, but provide too many fires to UFS‐Smoke on September 10th. As for the Pred and Pred2 cases, fire counts based on FIRA FRP predictions show a smoother diurnal variability (maximum change ∼ 100 count) compared to the Base and Fcst cases, probably due to the restricted spread area in the spread model discussed previously.

**Figure 14:**
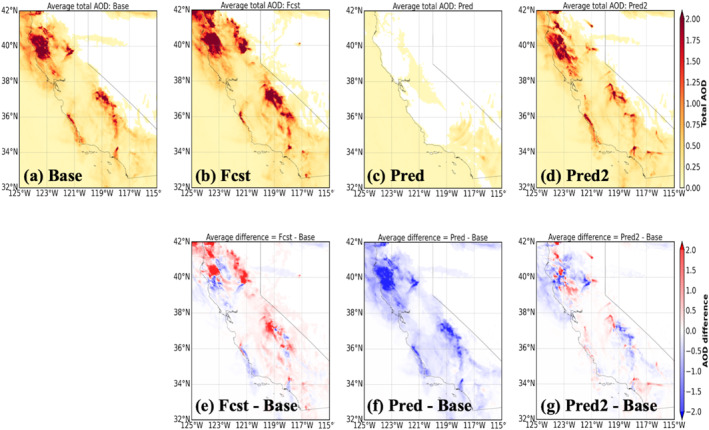
The average total‐column AOD during September 7th–10th, 2020 from the (a) Base, (b) Fcst, (c) Pred, and (d) Pred2 cases. The difference of average total‐column AOD of the (e) Fcst, (f) Pred and (g) Pred2 cases compared to the Base case.

**Figure 15:**
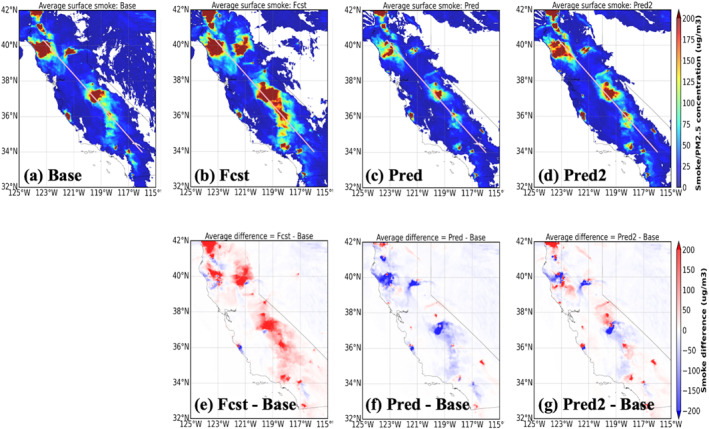
Same as Figure 14 but for average surface smoke concentration. The white lines shown in (a)–(d) are the location of the vertical cross‐section used in Figure 16.

**Figure 16:**
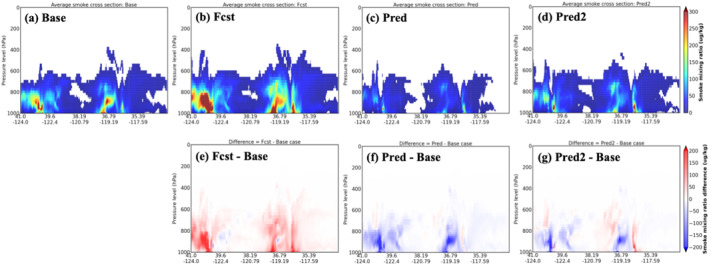
Same as Figure 14 but for the average vertical cross‐section of smoke mixing ratio. Location of the vertical cross‐section is marked as the white line in Figure 15.

Figures 14, 15, 16 illustrate the comparison of the total‐column aerosol optical depth (AOD), surface smoke concentration, and vertical cross‐section of smoke mixing ratio for the four UFS‐Smoke simulations, respectively. Location of the vertical cross‐section is marked as the white line in Figure 15. The three variables collectively provide a comprehensive picture of UFS‐Smoke results in a three‐dimensional scale. Table 6 is the spatial average of total‐column AOD and surface smoke concentration over the given domain and the average smoke mixing ratio over the selected cross‐section, along with the average differences between the Base case and the other three.

**Table 6:** Averages of Total‐Column AOD and Surface Smoke Concentration Over Selected Domain, and Average Smoke Mixing Ratio Over Selected Cross Section for the Four UFS‐Smoke Simulations, Along With the Difference of the Averages of Specific Parameters From the Fcst, Pred, and Pred2 Cases Relative to the Base Case

Simulation	Average	Average difference
Total‐column AOD		
Base	0.34 ± 0.54	–
Fcst	0.42 ± 0.83	0.10 ± 0.46
Pred	0.13 ± 0.04	−0.24 ± 0.40
Pred2	0.30 ± 0.49	**−0.02 ± 0.30**
Surface smoke mass concentration (ug m^−3^)		
Base	66.21 ± 248.71	–
Fcst	94.74 ± 362.93	17.29 ± 96.89
Pred	46.03 ± 167.46	−9.19 ± 44.07
Pred2	67.29 ± 264.09	**−0.72 ± 54.72**
Smoke mixing ratio cross section (ug kg^−1^)		
Base	41.62 ± 140.63	–
Fcst	69.20 ± 206.91	24.27 ± 45.73
Pred	18.29 ± 68.43	−18.24 ± 37.77
Pred2	33.71 ± 121.25	**−6.62 ± 33.18**

*Note*. The significantly improved average difference statistics for Pred2 compared to Fcst are shown in bold.

Overall, the Fcst case shows the highest AOD, surface smoke concentration, and vertical smoke mixing ratio with average differences of 0.10 ± 0.46, 17.29 ± 96.89 μg m^−3^, and 24.27 ± 45.73 μg kg^−1^ respectively, compared to the Base case. Results from the Pred case are significantly lower than the Base case, associated with the underestimation in FRP predictions in the intensity model. Because fire emissions are estimated based on FRP in UFS‐Smoke, underestimated FRP values will result in less smoke emissions and eventually lower AOD and smoke concentrations. In addition, low FRP values introduce a weak heat flux and a shallow smoke plume height (Freitas et al., 2007, 2010), which affects the location and advection of smoke emissions. The vertical cross‐section of smoke mixing ratio (Figure 16c) also shows that most smoke particles are suppressed near the surface in the Pred case due to a shallow smoke plume height when underestimated FRP predictions are used. On the other hand, the Pred2 case, which is based on the doubled FIRA FRP predictions, shows good agreement with the Base case and less systematic biases compared with the Fcst case. Total‐column AOD and surface smoke concentrations are higher near the fire sources and lower in the far downwind regions in the Pred2 case, leading to the lowest absolute differences comparing with the Base case (−0.02 ± 0.30 for AOD, −0.72 ± 54.72 μg m^−3^ for surface smoke concentration). This result is impacted by less intense smoke plumes that are transported across the state (Figure 16g) due to underestimated FRP values. Overall, although FIRA FRP prediction is generally underestimated, with simple adjustments in Pred2 it can better represent the fire behavior, spread, and impacts on smoke AOD compared with using fire persistence assumptions in the Fcst case.

### 4.2 Air Quality Forecast Application Assessment on Surface PM_2.5_ Concentrations

**Figure 17:**
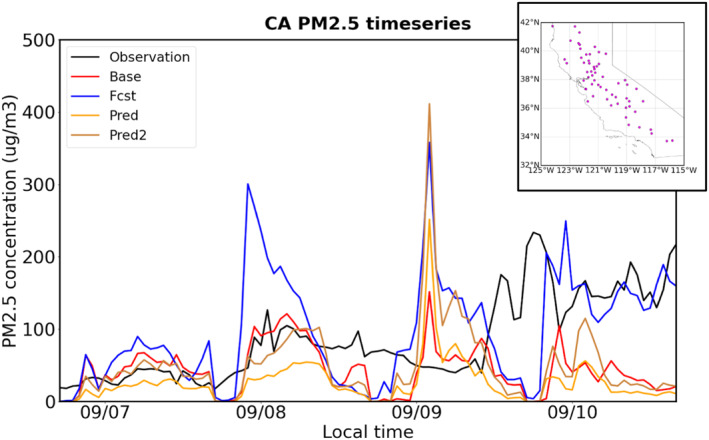
Timeseries of the average surface PM_2.5_ observations (black), and the average surface smoke concentrations from the Base (red), Fcst (blue), Pred (yellow), and Pred2 (brown) cases during September 7th–10th, 2020 at selected AirNow monitoring sites shown in the top‐right map.

In this section, the surface smoke concentrations from the four UFS‐Smoke simulations are compared to the surface PM_2.5_ observations from AirNow. Note that the AirNow PM_2.5_ concentrations shown here have been modified by removing the influence from local emissions following the baseline approach described in Section 2.1. Figure 17 is the hourly timeseries of the statewide average surface smoke/PM_2.5_ concentrations at selected monitoring sites in California during the fire period. On September 7th and 8th, the smoke concentrations from Base, Pred, and Pred2 cases show a good agreement with AirNow by capturing the trend of average PM_2.5_ concentrations associated with fire activities, while the smoke concentrations from the Fcst case are overestimated with a maximum average value about 300 μg m^−3^ on Sep 8th. However, on September 9th, all the UFS‐Smoke simulations introduce high smoke concentrations with maximum statewide averages over 400 μg m^−3^ and extremely high smoke concentrations exceeding 1,000 μg m^−3^ at the Yosemite Village site in central California, close to the Creek Fire, even for the Base and Pred cases. Given the common phenomenon in all simulations, the failure should be associated with the overall uncertainties in overestimated fire emissions within UFS‐Smoke instead of the choice of FRP inputs. As for September 10th, the smoke concentrations from the Fcst case align well with AirNow in general as those from the other three cases are significantly underestimated. According to the weather maps from the National Centers for Environmental Prediction (NCEP), a low pressure system moved into the Pacific Ocean and away from California on this day, following with increasing surface pressure and decreasing surface wind speeds near the fire regions (Appendix 8 in Supporting Information S1). Those meteorological conditions could suppress atmospheric dispersion and are favorable for particle accumulation, leading to the high PM_2.5_ concentrations measured at AirNow monitoring sites. Moreover, since the RAVE FRP data used in the Base case, which is based on near‐real‐time observations, is relatively low compared to the previous 3 days (Figure 12e), the contribution of fire emissions to PM_2.5_ concentrations should be minor. The high smoke concentrations from the Fcst case should be due to the overrepresented fire activities by using the FRP on September 9th for September 10th. Therefore, although the Fcst case shows a good agreement with AirNow, it still raises concerns about the representation of the FRP data from the previous day in operational applications.

**Figure 18:**
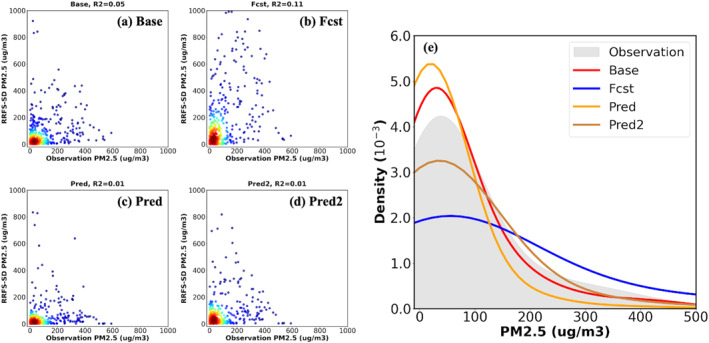
Comparison of surface smoke concentrations from the four UFS‐Smoke simulations and the PM_2.5_ concentrations at selected AirNow monitoring sites, including the density scatter plots of AirNow PM_2.5_ observations and UFS‐Smoke surface smoke concentrations for the (a) Base, (b) Fcst, (c) Pred and (d) Pred2 cases, and (e) the density histogram plot. In (a)–(d), colors show the counts of valid pairs of data points. In (e), PM_2.5_ observation is shown as gray shading patch while surface smoke concentrations for the Base, Fcst, Pred and Pred2 cases are shown as red, blue, yellow and brown lines, respectively.

Figure 18 is the site‐by‐site comparison of the surface smoke concentrations from the four UFS‐Smoke simulations and the PM_2.5_ concentrations, and Table 7 is the statistical analysis at all selected AirNow monitoring sites. First, all the four UFS‐Smoke simulations show nearly no correlation compared with AirNow with R^2^ about and even below 0.1 (Figures 18a–18d). However, the distribution of the smoke concentrations from the Base and Pred cases agrees well with the PM_2.5_ concentrations from AirNow (Figure 18e). Although the average smoke concentrations from the two cases are relatively low (∼55 μg m^−3^ for the Base case and 39 μg m^−3^ for the Pred case) compared to the average PM_2.5_ concentration from AirNow (∼70 μg m^−3^), the variances are comparable (12,620.87, 12,791.00, and 11,709.13 μg^2^ m^−6^ for AirNow, the Base case, and the Pred case, respectively). Furthermore, the smoke concentrations from the Pred2 case shows the lowest absolute MB (∼14 μg m^−3^) among the four UFS‐Smoke simulations but has relatively high variance (31,659 μg^2^ m^−6^) and RMSE (205 μg m^−3^) against the PM_2.5_ concentrations from AirNow, resulting in a slightly right‐skewed distribution (Figure 18e). This indicates that the Pred2 case generally captures the variabilities of surface smoke concentrations but tends to overestimate surface smoke concentrations for certain sites. On the other hand, the Fcst case, overpredicts the smoke concentrations with a positive MB (37.86 ± 234.07 μg m^−3^), the highest RMSE (237.11 μg m^−3^), and a significantly right‐skewed distribution with the highest variance (43,527.63 μg m^−3^). Overall, the Pred and Pred2 cases agree better with the Base case and AirNow compared with the Fcst case, demonstrating the enhanced reliability of FIRA FRP predictions for AQF applications.

**Table 7:** The Average Surface PM_2.5_ Observation and Surface Smoke Concentrations From the Four UFS‐Smoke Simulations at Selected AirNow Monitoring Sites, As Well As the Statistical Analysis (Variance, MB, *R* ^2^, and RMSE) of UFS‐Smoke Smoke Concentrations Compared to PM_2.5_ Observation

	Average (μg m^−3^)	Variance (μg^2^ m^−6^)	MB (μg m^−3^)	R^2^	RMSE (μg m^−3^)
AirNow	70.28 ± 112.34	12620.87	–	–	–
Base	55.68 ± 113.10	12791.00	−21.76 ± 136.71	0.05	138.43
Fcst	99.94 ± 208.63	43527.63	37.86 ± 234.07	0.11	237.11
Pred	39.00 ± 108.21	11709.13	−45.09 ± 158.33	0.01	164.63
Pred2	65.72 ± 177.93	**31659.09**	**−14.01 ± 204.53**	0.01	**205.01**

*Note*. The significantly improved average difference statistics for Pred2 compared to Fcst are shown in bold.

## 5 Model Limitations

As discussed in Section 3, the major uncertainties of FIRA are associated with the training data and underlying assumptions made to filter certain fires and achieve working ML models. Although summer 2020 was an intensive wildfire season and many weak and dying fires (isolated and with FRP <15 MW) are filtered out in data preprocess, the number of weak fires with FRP value less than 80 MW (∼66% of total data) is still double of that of strong fires with FRP value exceeding 80 MW (∼33% of total data). Since ML is a statistical approach trying to simulate the “common behavior” of the data set, the inclusion of many weak fires will negatively impact the ML model's ability to predict the extremely high FRP values, which often have less samples, and eventually lead to a general underestimation in model predictions. It is worth noting that training multiple spread and intensity models depending on different fire types does not noticeably improve model performance (not shown), probably because the ML models mainly predict the flammability of fuels. As discussed in Section 3.2 and Appendix 6 in Supporting Information S1, the connections between input variables and the physical mechanisms of fires spread are not fully represented, making it challenging for the ML models to identify the fire behaviors of different fire types.

The limitation of the ability of satellite sensors to observe an adequate number of nighttime fires does not provide sufficient information of fire diurnal variabilities to the ML models, which further degrades the performance of multi‐hour forecast by creating overactive fire activities during nighttime. In addition, a lack of variability of ST is found as some STs show relatively low frequency in the training data (Appendix 1 in Supporting Information S1), which may be due the distribution of natural ecosystem. For instance, evergreen broadleaf (ST = 2) and deciduous needleleaf/broadleaf forests (ST = 3 and 4) are relatively rare as most forests in CONUS are identified as mixed forests (ST = 5). Including more training data with extended study period and domain, and additional data adjustments such as manually increasing the number of high FRP cases could be possible solutions. Also, improving the ML models' capabilities of learning the physical processes of fires spread, for example, by refining necessary data processes and adding weights to mereological drivers, could help the ML models distinguish daytime and nighttime fire behaviors.

Moreover, the definition of dying fires is relatively simplified. The isolated fires with low FRP values could, on the contrary, indicate the early stage of fire activities and should be considered in training data given their capabilities of spreading. Agricultural and grassland fires, which are also associated with low FRP values, could be misidentified as dying fires as shown in model evaluation (Section 3). Since extinguishment is a necessary stage for all kinds of fires regardless of the fire and fuel types, it is hard for the ML models to distinguish weak but lasting fires from dying fires based on given parameters (e.g., FRP, meteorology and fuel characteristics). Although FIRA successfully captures the overall trend of identified existing fires, it may fail to predict the correct extinguishment time by, for example, introducing a longer burning period.

To rectify this issue, adding the isolated weak fires back to the training data and improving the representation of meteorological parameters that contribute to fire behaviors (e.g., T and WS) in the ML models would be helpful. Information of fuel consumption and/or burned area, retrieved from estimated consumption rates (Wooster et al., 2005) for example, could also potentially inform the ML models of fire extinguishment. Fires at certain grid cells would cease out when most fuels within that region are nearly consumed. Furthermore, additional fire phase (e.g., flaming and smoldering) information is potentially available from satellite measurements. Recent studies have demonstrated that the carbon monoxide (CO) and nitrogen dioxide (NO_2_) measurements from the Ozone Monitoring Instrument (OMI) and TROPOspheric Monitoring Instrument (TROPOMI) can be used for the quantification of fire combustion efficiency from space (Lama et al., 2020; Silva & Arellano, 2017; W. Tang & Arellano, 2017; van der Velde et al., 2021). High combustion efficiencies reflect active fires with more flaming burns, while low combustion efficiencies are usually associated with smoldering, dying fires. However, TROPOMI and OMI are polar‐orbiting satellites and provide data on a daily basis, which may not properly represent the real‐time fire characteristics especially for agricultural fires that only last for hours. The hourly products from the recently launched geostationary satellite, the Tropospheric Emission: Monitoring of Pollution (TEMPO), could potentially provide fire phase information to the ML models for a better performance in the prediction of weak fires.

In addition, assumption of a maximum rate of spread in the data preprocess stage is another major source of uncertainties in fire spread prediction. The spread model is not able to capture the full scope of rapid‐spreading fires (e.g., big wildfires with spread rates of 10–30 km hr^−1^ [Liu et al., 2021]) due to restricted spread area (15 × 15 km^2^ in this study). This could be solved by increasing the size of data frames for better coverage.

It is also worth noting that the uncertainties of the sources of input variables (i.e., HRRR and satellite products) and the sensitivities of the ML models to different input data sets are not considered in this study. Since ML is a data driven approach, the choice of input data set may introduce considerable impacts on the performance of ML models. The data sets described in Section 2.1 are selected in an operational‐forecast manner as an example for the demonstration of FIRA's capability, and could be replaced based on users' interests and applications.

Lastly, FIRA focuses on the propagation of existing fires and does not consider new ignitions. Since fire‐fire interactions can affect the spread of fires within the region (Collins et al., 2009; Finney & McAllister, 2011), missing new ignitions may introduce uncertainties to the fire prediction to some extent. Many fire indices such as the fire weather index (FWI; Di Giuseppe et al., 2016, 2017) and ML‐based models (Jain et al., 2020) have developed for the predictions of fire risk/fire occurrence. Coupling with these applications could potentially improve the accuracy of FIRA multi‐hour forecast by adding the information of new ignitions to the ML models, while potentially also mitigate other uncertainties described above (e.g., defining dying vs. early fire activities).

## 6 Conclusions

This study develops a machine learning (ML) based fire spread forecast system, Fire Intensity and spRead forecAst (FIRA), that provides the predictions of the spatial distribution and intensity of fire radiative power (FRP) used widely in Air Quality Forecast (AQF) models. Two ML models are developed to predict the fire spread and intensity in the next hour, and a forecast workflow is generated for multi‐hour forecasts by feeding model predictions into forecast cycles. Results show that FIRA well captures the spread of fires with good spatial similarity (∼95% for the next hour forecast and >60% for 24 hr forecast). However, negative‐based FRP values are found, indicating a general underestimation of fire intensity in FIRA. In addition, FIRA has difficulties in predicting nighttime fires and the extinguish time in multi‐hour forecasts, mainly associated with uncertainties in the training data and underlying assumptions.

Sensitivity analysis of the experimental UFS coupled with smoke (UFS‐Smoke) model simulations with real‐time FRP products from the Regional ABI and VIIRS fire Emissions (RAVE) and FIRA FRP predictions are conducted and used as the assessment of an AQF application. A modification of FIRA FRP by scaling the FRP values by a factor of 2 is also applied as an example of a potential solution to the underestimated FRP values. Overall, the UFS‐Smoke simulations using RAVE FRP and modified FIRA FRP prediction show a good agreement with the average differences of −0.02 ± 0.30 for aerosol optical depth (AOD) and −0.72 ± 54.72 μg m^−3^ for surface smoke concentration. Moreover, the surface smoke concentrations from the UFS‐Smoke simulation using the modified FIRA FRP prediction align well with the fire‐affected PM_2.5_ observations at selected AirNow monitoring sites with a mean bias around −14 μg m^−3^. These results indicate that FIRA FRP predictions can represent the future fire activities properly in terms of fire locations and the underestimation can be improved by using simple scaling factors. However, current FIRA is mainly applied for larger fires such as wildfires in the western US that usually last for multiple hours and even days. Most small prescribed fires, which are common in the eastern and southeastern US, only stay for a couple of hours on a particular day and may not spread over a large area across multiple grid cells. Without the information of new ignitions and with isolated small fires removed, FIRA is limited to predicting the future of such short‐burning prescribed fires in multi‐day forecasts.

To the best of our knowledge, FIRA is the first ML‐based fire spread forecast model that generates predictions of fire location and intensity simultaneously. Since the two ML models (i.e., the spread and intensity models) are trained independently, they can be used as standalone model applications and have the flexibility to be coupled with different AQF model systems. The data preprocessor and fire mapper described in this manuscript are examples of the essential data processing steps and could be replaced by the built‐in data processors in coupled forecast systems such as the Unified Forecast System (UFS). Moreover, the spread model alone could also be used for short‐range fire alarms and provide valuable information of the forward direction of fires to fire fighters and government agencies for better resource arrangement and damage control.

Future improvements include refinement of assumptions (e.g., definition of dying fires and calculation of modified VHI) and additional input variables such as soil moisture, fuel moisture and downward shortwave radiation to better represent the land surface characteristics and fire behaviors. Estimation of fuel consumption and/or combustion efficiency are also expected to improve the ML models' capability of capturing fire extinguishment. Satellite‐based leaf area index (LAI) measurements, and high‐resolution vegetative canopy height products are considered to improve the representation of fuel characteristics in the ML models. Firefighting operations would also be considered for the influence of human activities and fire controls. Adjustments in model structure and configuration are expected, such as an extended spread area (e.g., from 15 × 15 km^2^ to 30 × 30 km^2^) and extra weights to meteorological parameters, to improve the representation of the physical processes of fire activities and spatial correlation between grid points. While this study focuses on the CONUS domain and fire activities in 2020, a longer time period of multiple years and an extended domain of North America based on the latest RAVE version 2 are considered. Finally, global application could be available by leveraging the hourly FRP data from various instruments around the globe (e.g., himawari‐8).

## Conflict of Interest

The authors declare no conflicts of interest relevant to this study.

## Supporting information

Supporting Information S1

## Data Availability

The source code of the canopy model set is publicly available at Hung and Moon (2024). The data sets used in this study are available to download (some per request) from the following links. Abbreviation and labels are referred to Table 1. RAVE^a^ https://sites.google.com/view/rave‐emission/ HRRRv3^b^ https://home.chpc.utah.edu/~u0553130/Brian_Blaylock/cgi‐bin/hrrr_download.cgi. VIIRS AST^c^ https://www.star.nesdis.noaa.gov/jpss/st.php. VIIRS VHP^d^ https://www.star.nesdis.noaa.gov/smcd/emb/vci/VH/vh_ftp.php. GEDI + Landsat^e^ https://glad.umd.edu/dataset/GLCLUC2020. Yamazaki et al. (2017)^f^ https://github.com/CliMA/GriddingMachine.jl. RAP^g^ https://www.ncei.noaa.gov/products/weather‐climate‐models/rapid‐refresh‐update. AirNow^h^ https://aqs.epa.gov/aqsweb/documents/data_api.html.
