# Supplementary material for: Fire Intensity and spRead forecAst (FIRA): A Machine Learning Based Fire Spread Prediction Model for Air Quality Forecasting Application
Source: Geohealth. 2025 Mar 22;9(3):e2024GH001253. doi: 10.1029/2024GH001253 (PMC11928747; doi:10.1029/2024GH001253)
Supplement: Supplementary file 1 — Supporting Information S1 [file GH2-9-e2024GH001253-s001.pdf]

Fire Intensity and spRead forecAst (FIRA): A machine learning based fire spread prediction model for air quality forecasting application

Wei-Ting Hung[1,2,3,*], Barry Baker[1], Patrick C. Campbell[1,2,3], Youhua Tang[1,2,3], Ravan Ahmadov[4], Johana Romero-Alvarez[4,5], Haiqin Li[4,5], Jordan Schnell[4,5]

[1]Air Resources Laboratory, National Oceanic and Atmospheric Administration, MD, USA

[2]Cooperative Institute for Satellite and Earth System Studies, University of Maryland, MD, USA

[3]Center for Spatial Information Science and Systems, George Mason University, VA, USA

[4]Global Systems Laboratory, National Oceanic and Atmospheric Administration, CO, USA

[5]Cooperative Institute for Research in Environmental Sciences, University of Colorado, CO, USA

*Correspondence: Wei-Ting Hung <wei-ting.hung@noaa.gov>

Contents

Supplementary Figures A1 to A6

Supplementary Tables A1 to A4

Supplementary Materials:

- Appendix 1: Distribution of input variables used in the training process.
- Appendix 2: Preliminary analysis of modified vegetation health index.
- Appendix 3: Configurations of the machine learning models.
- Appendix 4: Sensitivity test of the threshold of the possibility of fire occurrence used for fire spread prediction.
- Appendix 5: Definitions of statistical scores used for model evaluation.
- Appendix 6: Investigations of the contributions of non-FRP parameters in machine learning models.
- Appendix 7: Spatial distribution of fire radiative power for the Park Fire.
- Appendix 8: Analysis of the synoptic weather on September 9th - 10th, 2020.

Appendix 1

The distribution of the 12 input variables used in the training process is shown as Figure A1, including fire radiative power (FRP), terrain elevation (ELV), surface type (ST), day of year (DOY), hour of the day (HOUR), forest height (FH), vegetation health index (VHI), 2-meter temperature (T), 2-meter specific humidity (SH), precipitation rate (PRATE), and 10-meter wind speed and direction (WS and WD). More details can be found in Section 2.1 and Table 1.

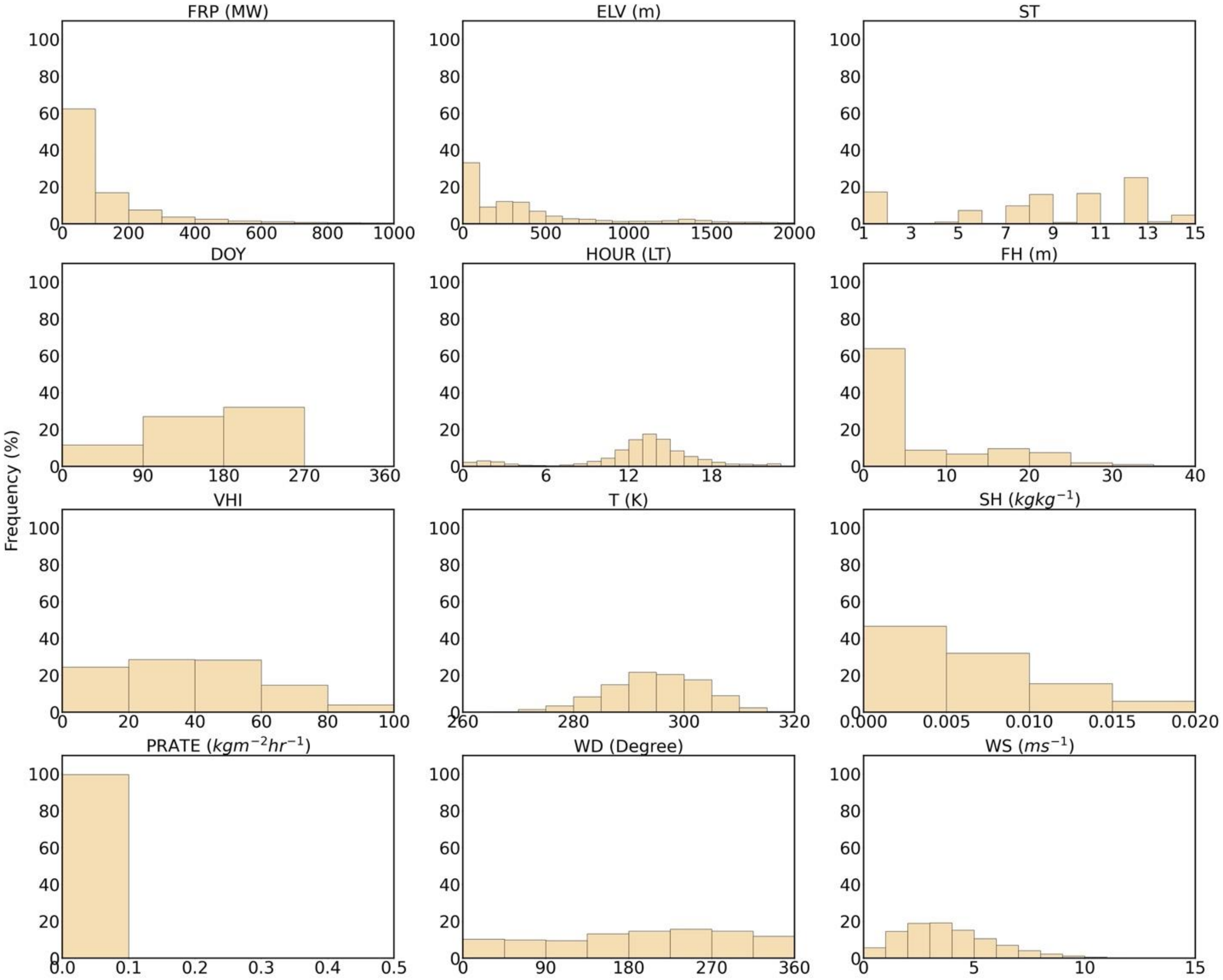

Figure A1. Density histogram plots of the 12 input variables used in the training process.

Appendix 2

Figure A2 shows preliminary results of the comparisons of the modified vegetation health index (VHI) defined as Eq. 1 and the standard VHI. While consistently negative differences are found over CONUS, VHIs from both definitions (30-to-70 partition v.s. 50-to-50 average) show similar regional and seasonal variabilities. Therefore, their influence on vegetations and fire behaviors are highly correlated, and the negative differences between them should not affect the training results noticeably. However, the 30-to-70 partition used in Eq. 1 is not validated without explicit sensitivity tests and additional works are required.

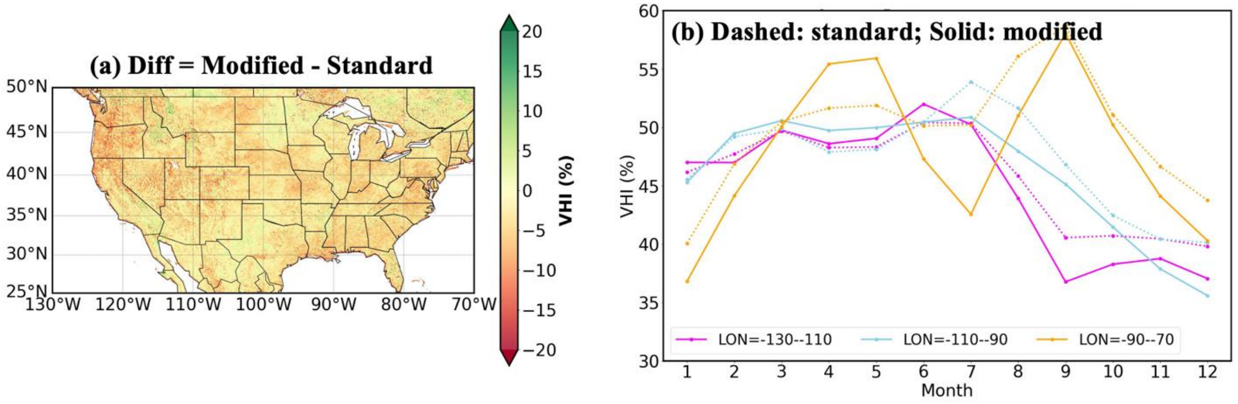

Figure A2. The (a) annual average difference between the modified and standard VHIs over CONUS, and (b) monthly average of the modified and standard VHIs in different meridian regions.

Appendix 3

The training algorithms and hyperparameters used in the spread and intensity models are given in Table A1 and A2, respectively. In the spread model, a customized loss function, in which grids with zeros are assigned a loss weight as 0.6 while others are assigned as 1, and a learning rate scheduler, which reduces the learning rate by 30% every 100 epochs, are used to avoid overfitting. In the intensity model, the hyperparameters are determined by using the hyperparameter tuning tool GridSearchCV from the Python scikit-learn package (Pedregosa et al., 2011; https://scikit-learn.org/stable/modules/generated/sklearn.model_selection.GridSearchCV.html).

Table A1. Configurations of the spread model.

Algorithm/Hyperparameter	Configuration
Activation function	LeakyReLU/Sigmoid
Optimization function	Adamax
Epoch #	300
Batch #	100
Neuron #	20
Kernel size	3
Pool size	2
Dropout rate	0.5
Initial learning rate	0.0001

Table A2. Configurations of the intensity model.

Algorithm/Hyperparameter	Configuration
Optimization	Histogram optimizer
Loss metric	Root mean square error
Max tree depth	10
# of parallel trees	10
Iteration/# of estimator	200

Subsample ratio	0.6
Learning rate	0.1

Appendix 4

To determine the threshold of the possibility of fire occurrence used for the prediction of fire spread, a sensitivity test is conducted by examining the frame similarity (FS) for the predictions of the spread model based on possibility threshold from 5% to 40% with an interval of 5%. Results are shown in Table A3. The optimal value for such threshold is assumed to be between 20 – 30%, while the FS for agricultural/grassland fires peaks when possibility threshold equal to 20% and the FSs for all fire types and wildfires are nearly consistent when possibility threshold over 30%. A loose threshold (possibility of 20%) is used in this study to get a better presentation for agricultural/grassland fires.

Table A3. Frame similarity for the predictions from the spread model based on possibility threshold from 5% to 40% with an interval of 5% for all fires, wildfires and agricultural/grassland fires during August 2020.

Possibility threshold	Frame similarity (%)		
	All	Wildfire	Agricultural/Grassland
5%	88.93 ± 13.99	83.75 ± 15.86	94.25 ± 9.08
10%	92.71 ± 9.59	89.68 ± 11.30	95.82 ± 6.05
15%	93.87 ± 8.21	91.57 ± 9.81	96.24 ± 5.17
20%	94.13 ± 7.73	91.86 ± 9.26	96.46 ± 4.73
25%	94.27 ± 7.63	92.26 ± 9.15	96.33 ± 4.90
30%	94.31 ± 7.56	92.33 ± 9.03	96.33 ± 4.89
35%	94.33 ± 7.50	92.38 ± 8.97	96.34 ± 4.87
40%	94.34 ± 7.50	92.39 ± 8.96	96.34 ± 4.86

Appendix 5

For the binary frame predictions from the spread model, frame similarity (FS) is calculated as:

$$FS = \frac{N_{match}}{N_{total}} \times 100\% \qquad \text{Eq. A1}$$

where N_{match} is the number of the consistent grids of each pair of binary frames from observation and model prediction, and $N_{total} = 25$ is the total number of grids in each frame. FS equals to 100% when the data frames from observation and model prediction are identical.

As for the intensity model, since the FRP frame predictions contain non-binary values, they need to be binarized before calculating FS. To keep both the location and intensity information, a modified average hashing technique (Hamadouche et al., 2021) is performed. First, the FRP values in each frame are normalized and the average of normalized values is then calculated. Second, hashes are generated by comparing the normalized values with the average. Figure A3 shows an example of the hash generation process. Grids with normalized values above and below the average are identified as ones and zeros in the hashes, respectively. Finally, the hashes will be used for FS calculations (Eq. A1). For the example shown in Figure 2, $N_{match} = 24$ with 24 consistent grids between two hashes and FS is 96%. It is worth noting that, since the hashing technique not only considers the location of valid grids but also accounts for the relative distribution of low/high FRP values, it is possible that the intensity model provides a slightly different FS compared to the spread model.

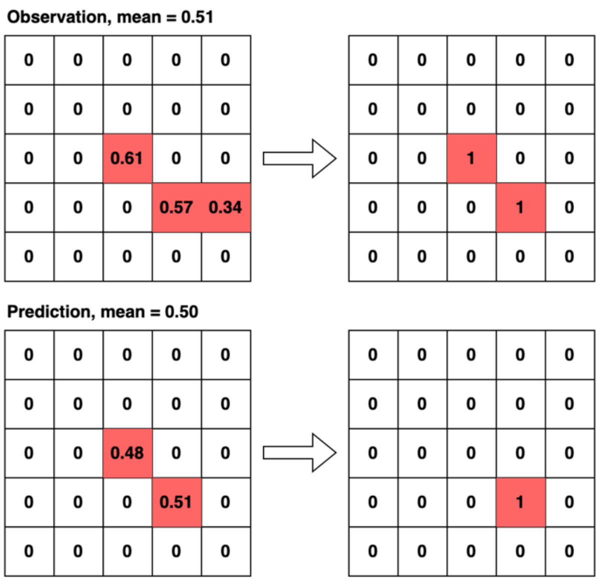

Figure A3. Example of average hashing for frame similarity calculation.

Three additional statistical scores are used for the evaluation of the prediction of fire intensity (i.e. the FRP values) generated by the intensity model, including mean bias (MB), coefficient of determination (R^2), and root mean square error (RMSE). The scores are calculated as:

$$MB = \frac{1}{n}\sum_{i}^{n} Y_i - X_i \quad \text{Eq. A2}$$

$$R^2 = \left(\frac{\sum_i^n (Y_i - \bar{Y}) \sum_i^n (X_i - \bar{X})}{\sqrt{\sum_i^n (Y_i - \bar{Y})^2} \sqrt{\sum_i^n (X_i - \bar{X})^2}}\right)^2 \quad \text{Eq. A3}$$

$$RMSE = \sqrt{\frac{1}{n}\sum_{i}^{n} (Y_i - X_i)^2} \quad \text{Eq. A4}$$

where n is the number of data points, X is observation, Y is prediction, and $\bar{X}$ and $\bar{Y}$ are the averages of observation and prediction, respectively. The statistical scores and FS of each binary/FRP frame predictions in test data are then averaged as estimated model scores.

The alarm rates of true alarms, false alarms and miss alarms are calculated as the measures of good forecast, false recognition, and missing case, respectively and used to evaluate the accuracy of

gridded FRP prediction generated by the fire mapper. The alarm rates are defined as the ratios of the number of true/false/miss forecasts to the number of total forecasts. The calculation focuses on the defined spreading domain of 15 x 15 km^2 in the spread model.

Furthermore, to understand the relationships between input variables and predictions from the ML models (i.e. the fire spread and FRP predictions), the permutation variable importances (VIs; McGovern et al., 2019) of 12 input variables are estimated for the spread and intensity models. VI describes the impacts of selected input variables on model performance when breaking the correlations between the input variables and model outputs. It is estimated by randomly shuffling the variable of interest in test data while keeping the others unchanged, and comparing the statistical scores of the shuffled model and unshuffled model. This process is repeated until all input variables have been shuffled once. In this study, the percentage error of the RMSE values of the shuffled and unshuffled models is calculated as the estimation of VI. Larger percentage errors indicate higher VIs.

$$VI = \frac{RMSE_{shuffled} - RMSE_{unshuffled}}{RMSE_{unshuffled}} \times 100\% \qquad \text{Eq. A5}$$

The three statistic scores described above (i.e. MB, R^2 and RMSE) of $PM_{2.5}$ observation from selected AirNow monitoring sites and surface smoke concentration from the four UFS-Smoke simulations are analyzed for the assessment to AQF model application. The variances of AirNow $PM_{2.5}$ observation and UFS-Smoke surface smoke concentrations are also calculated:

$$Variance = \frac{1}{n}\sum_{i}^{n}(X_i - \bar{X})^2 \qquad \text{Eq. A6}$$

where n is the number of data points, X is the data of interest (e.g. $PM_{2.5}$ and/or smoke concentrations), and $\bar{X}$ is the average of X.

Appendix 6

To better verify the reasons of low variable importance (VI) of non-FRP parameters (e.g. T and WS) in the ML models, two additional sets of ML models (i.e. the spread model plus the intensity model) are trained based on the same model configurations described in Section 2 but with different input variables. For the FRP-only case, the ML models are trained by using FRP only, while the ML models are trained by all parameters except for FRP for the FRP-excluded case.

Table A4 shows the model statistical results of the model predictions from two cases compared to RAVE FRP for all fires, wildfires and agricultural/grassland fires during August 2020. Results showed that the performance of the ML models from the FRP-excluded case significantly degraded, which is expected as FRP shows dominant contributions to the ML models (Section 3.2). However, the model performance of the FRP-only case is relatively identical with the results discussed in Section 3, while average RMSE slightly increases by ~ 3% for all fires. Differences are more noticeable for agricultural/grassland fires with increases by 30 – 40% in MB and RMSE are found, indicating that non-FRP parameters still affect model performance to some extent despite the low VIs. One major concern is aliasing effect, which often happens when performing data downsampling (Ribeiro & Schön, 2021; Vasconcelos et al., 2021). When data resolution is too low, the original spatial variability of given dataset may become indistinguishable from the downsampled data in the ML models. Another possibility is the internal interactions among and/or combined contribution from non-FRP parameters. For instance, terrain slope and wind speed collectively affect the rate of fire spread (Rothermel, 1972). Ranking VI cannot reveal such interactions since VI focuses on the 1-to-1 correlation between input variable and model prediction.

Moreover, the ranking VIs of the spread and intensity models from the FRP-excluded case is illustrated in Figure A4. VHI and ST become the dominant contributors in the spread model and

ST becomes dominant in the intensity model compared to the results shown in Figure 5. The differences may indicate that the ML models are mainly predicting the flammability of vegetations/fuels instead of the physical movement of fire activities. In addition, the decreased VI rank of WD implies that the ML model could not learn the spreading direction of fires without provided the initial location of fires.

Table A4. Statistical results of the predictions from spread and intensity models in the FRP-only and FRP-excluded cases compared to RAVE FRP for all fires, wildfires and agricultural/grassland fires during August 2020.

	All	Wildfire	Agricultural/Grassland
	FRP-excluded		
FS – Spread model (%)	82.53 ± 18.23	75.57 ± 19.15	89.66 ± 14.01
FS – Intensity model (%)	83.03 ± 16.80	76.32 ± 17.48	89.90 ± 12.84
MB (MW)	-2.65 ± 71.58	-17.40 ± 101.90	12.45 ± 28.03
R^2	0.30 ± 0.33	0.21 ± 0.27	0.45 ± 0.37
RMSE (MW)	93.13 ± 160.12	156.79 ± 202.98	27.92 ± 35.99
	FRP-only		
FS – Spread model (%)	94.18 ± 7.78	92.10 ± 9.32	96.31 ± 4.97
FS – Intensity model (%)	94.63 ± 6.75	92.88 ± 7.96	96.40 ± 4.59
MB (MW)	-5.65 ± 41.87	-14.25 ± 56.08	3.15 ± 13.18
R^2	0.72 ± 0.36	0.64 ± 0.36	0.83 ± 0.31
RMSE (MW)	58.21 ± 129.86	99.92 ± 165.80	15.47 ± 48.69

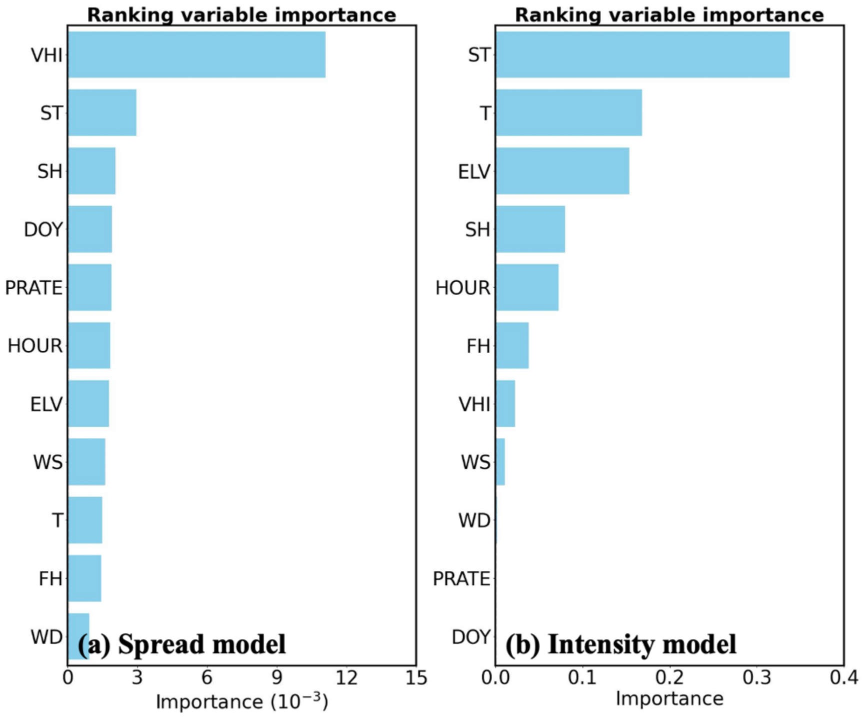

Figure A4. Ranking variable importance of training parameters used in the (a) spread model and (b) intensity model for the FRP-excluded case.

Appendix 7

The Park Fire experienced an aggressive growth after ignitions due to strong winds and massive dry fuels according to the status reports from the California Department of Forestry and Fire Protection (CAL FIRE) and satellite fire detections. Apparently, the rapid spreading exceeded the maximum rate of spread assumed in the ML models (Figure A5). Although the hot spots with the highest FRP values (e.g. red dots) move along with the spread direction of fires for early forecast times, the fire frame identified by FIRA does not capture the actual fire propagation after forecast time 12 h. In addition, the persistently burning fires generated by FIRA workflow suggest the potential needs of an indicator of fire extinguishment.

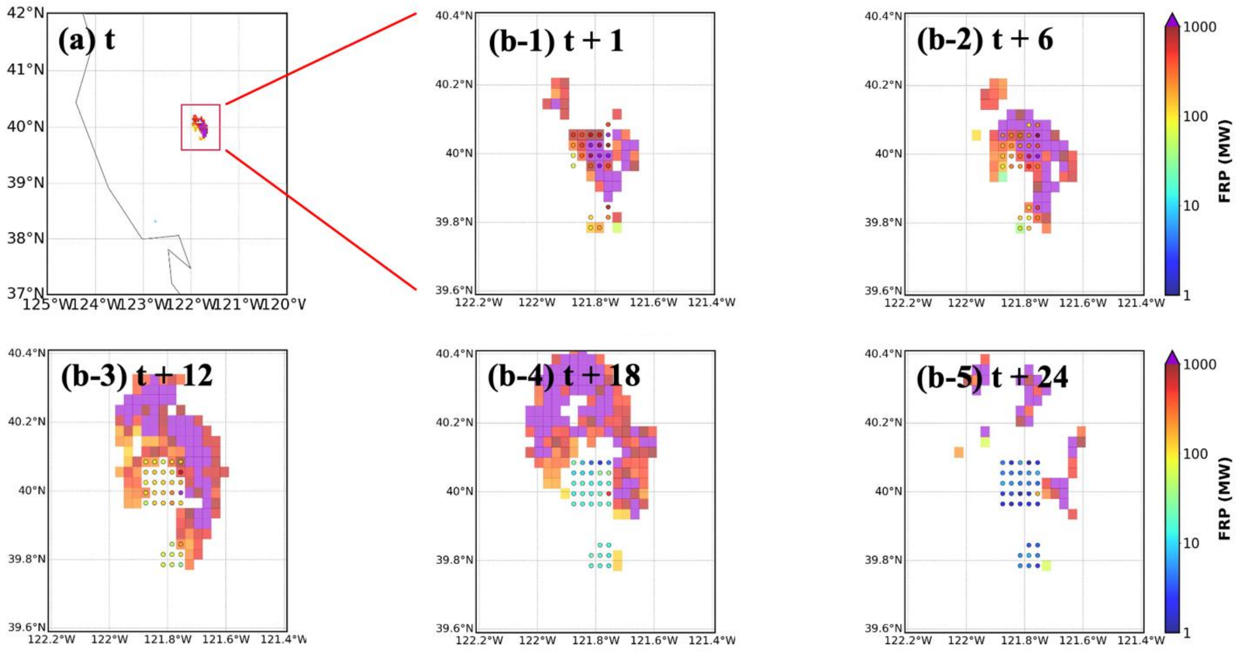

Figure A5. Spatiotemporal variation of RAVE FRP (shaded) at (a) initial time, and RAVE FEP covered by FIRA FRP (dots) at (b) forecast time 1 h, 6 h, 12 h, 18 h, and 24 h for the California

Park Fires on July 26th, 2024. The red box in (a) demonstrates the location of the selected fire incident. Same as Figure 11 while including fires outside the identified fire frame.

Appendix 8

According to the daily weather maps from the National Centers for Environmental Prediction (NCEP), a low pressure system moved away from the land toward the Pacific Ocean, resulting in higher surface pressure levels over California on September 10th, 2020. Similarly, HRRRv3 also shows an increasing trend in surface pressure from September 9th to 10th, along with a significant reduction in surface wind speeds. These phenomena are favorable for the accumulation of air pollutants with suppressed advection and could lead to high surface $PM_{2.5}$ concentrations.

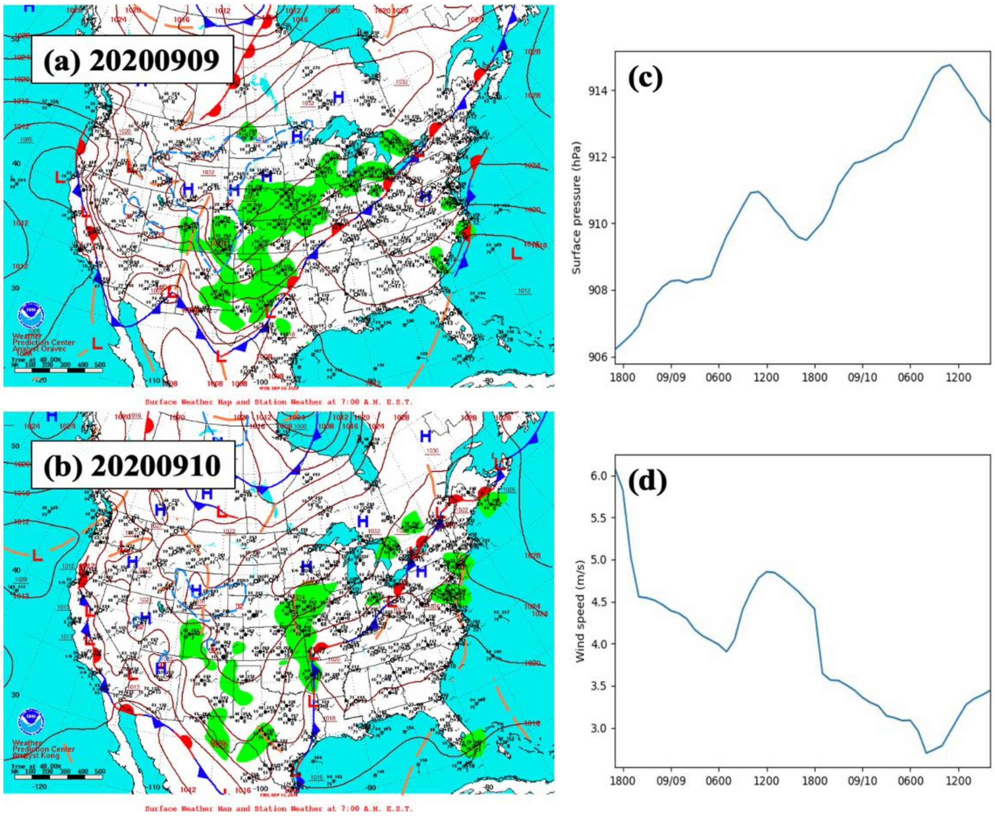

Figure A6. The daily weather maps on (a) September 9th and (b) September 10th, 2020. The timeseries of (a) surface pressure and (b) 10-m wind speed from HRRRv3 during September 9th – 10th averaging over the California domain shown in Figure 12.
